# Holistic Sustainability Assessment of Riparian Buffer Designs: Evaluation of Alternative Buffer Policy Scenarios Integrating Stream Water Quality and Costs

**DOI:** 10.3390/su141912278

**Published:** 2022-09-27

**Authors:** Santosh R. Ghimire, Adam C. Nayak, Joel Corona, Rajbir Parmar, Raghavan Srinivasan, Katie Mendoza, John M. Johnston

**Affiliations:** 1 U.S. Environmental Protection Agency, Office of Research and Development, Athens, GA 30605, USA; 2 Department of Civil and Environmental Engineering, Stanford University, Stanford, CA 94305, USA; 3 U.S. Environmental Protection Agency, Office of Water, Washington, DC 20460, USA; 4 Department of Ecology and Conservation Biology, Texas A&M University, Temple, TX 76502, USA

**Keywords:** riparian buffer zone, data envelopment analysis, watershed, sustainability, net present value cost

## Abstract

Riparian buffer zones (RBZs) have been shown to be effective best management practices (BMPs) in controlling non-point source pollutants in waterbodies. However, the holistic sustainability assessment of individual RBZ designs is lacking. We present a methodology for evaluating the holistic sustainability of RBZ policy scenarios by integrating environmental and economic indicators simulated in three watersheds in the southeastern USA. We developed three unique sets of 40, 32, and 48 RBZ policy scenarios as decision management objectives (DMOs), respectively, in Back Creek, Sycamore Creek, and Greens Mill Run watersheds (Virginia and North Carolina) by combining the RBZ—widths with vegetation types (grass, urban, naturalized, wildlife, three-zone forest, and two-zone forest). We adapted the RBZ—hydrologic and water quality system assessment data of instream water quality parameters (dissolved oxygen, total phosphorus, total nitrogen, total suspended solids—sediment and biochemical oxygen demand) as environmental indicators, recently published by U.S. EPA. We calculated 20-year net present value costs as economic indicators using the RBZ’s establishment, maintenance, and opportunity costs data published by the Natural Resources Conservation Service. The mean normalized net present value costs varied by DMOs ranging from 4% (grass RBZ—1.9 m) to 500% (wildlife RBZ—91.4 m) across all watersheds, due primarily to the width and the opportunity costs. The mean normalized environmental indicators varied by watersheds, with the largest change in total nitrogen due to urban RBZs in Back Creek (60–95%), Sycamore Creek (37–91%), and Greens Mill (52–93%). The holistic sustainability assessments revealed the least to most sustainable DMOs for each watershed, from least sustainable wildlife RBZ (score of 0.54), three-zone forest RBZ (0.32), and three-zone forest RBZ (0.62), respectively, for Back Creek, Sycamore Creek, and Greens Mill, to most sustainable urban RBZ (1.00) for all watersheds.

## 1. Introduction

Section 303(d) of the Clean Water Act (CWA), the primary law regulating pollution in waterways of the United States of America, requires states, territories, and authorized tribes to submit a list of impaired waters that are not meeting water quality standards for nutrients, sediments, and other unhealthy pollutants to the U.S. Environmental Protection Agency (EPA) for approval [1]. The U.S. EPA Office of Water is responsible for implementing the CWA, ensuring that the nation’s watersheds and waterbodies and their aquatic ecosystems are restored and maintained. The use of riparian buffer zones (RBZs) is considered a best management practice (BMP) for sustainable watershed management and is generally supported by the state and local governments as well as by broad stakeholders within and outside the U.S. RBZs provide multiple watershed ecosystem services by controlling non-point source pollutants in streams, lakes, and wetlands, improving biodiversity, and enhancing woodland connectivity corridors [2–5]. In the United States, various riparian restoration and preservation programs exist under the 1996 Farm Bill that include the continuous Conservation Reserve Program’s (CRP) Conservation Reserve Enhancement Program; the CRP is a voluntary land conservation program in which farmers can offer land, in return with rental payments and cost-share assistance, to establish long-term (10 to 15 years) to permanent resource-conserving plant species, such as approved grasses or trees [6,7].

Globally, several studies evaluated the RBZs’ effectiveness on controlling the sediments and nutrients and reported that the effectiveness varied across geographic regions [4,8–11], from 20% to 100% of nitrogen [9], 27% to 97% total phosphorous [12], and 84% to 90% sediment trapping effectiveness [13,14] across European countries to the southeastern United States. Recently, Ghimire et al. [14] performed a sensitivity assessment of RBZ design strategies to stream water quality in three southeastern U.S. watersheds and reported comparable reductions of sediment (61% to 96%), total nitrogen (34% to 55%), and total phosphorous (9% to 48%) as compared to the stream water quality without the RBZ applications.

Others assessed RBZ cost efficiencies [15–17] considering establishment, maintenance, and opportunity costs (i.e., foregone revenue due to inclusion of land in a RBZ) that varied by the type of buffer (e.g., variable width and vegetation) and land use type [16–21]. A synthesis of literature relevant to RBZ cost assessment is provided in Appendix A Table A1. To highlight, Trenholm et al. [15] estimated forestland opportunity costs and benefits of non-market ecosystem services (water filtration, wildlife habitat, and aesthetics) for riparian programs in a Canadian watershed using contingent valuation method (willingness-to-pay). Tiwari et al. [21] compared the opportunity cost of variable width RBZs to that of fixed width RBZs in a Swedish watershed. Qiu and Dosskey [20] presented a cost-effectiveness index aggregated with multiple benefits (e.g., impacts on wildlife habitat and stormwater runoff) of conservation buffer placement strategies (the riparian, the topography, and soil survey-based strategies) within a watershed in New Jersey (USA).

However, a gap exists in the understanding of a holistic sustainability of individual RBZ designs that integrates the economic and environmental pillars of sustainability. Watershed managers and planners frequently face practical difficulty to assess the RBZ’s holistic sustainability and to make an informed decision considering available resources and environmental (stream) water quality impacts. Sustainability of a system, society, or a product involves three pillars of economic, social, and environmental goals seeking answers to four questions: what to sustain, for whom, for how long (the current generation and future generations), and at what cost [22–24]. Thus, the holistic sustainability assessment of a system is challenging due to the involved multidisciplinary indicators, the complexity of environmental changes, social and economic issues, and subjectivity of the stakeholders’ views on the weights of the pillars of sustainability [24,25].

Select methods are available elsewhere for quantifying sustainability indicators and estimating integrated sustainability scores [24,26]. Ghimire and Johnston [24] presented a modified eco-efficiency framework comprised of economic, environmental, and social indicators that was applied to evaluate sustainability of agricultural systems and rainwater harvesting systems tailored to the Albemarle-Pamlico River basin (USA) [26]. They [24,26] used the International Organization for Standardization’s (ISO) life cycle assessment (LCA) approach for quantifying environmental indicators [27–32] and the life cycle cost assessment (LCCA) [33] approach for calculating economic indicators, and used data envelopment analysis (DEA), a widely applied statistical approach, to calculate the integrated sustainability scores in terms of modified eco-efficiency measures [34,35]. Pioneered by Farrell [34] and Charnes et al. [35], DEA was applied widely across the disciplines, for example, in assessing the eco-efficiencies of rainwater harvesting systems used in water resource management [24,26], industrial systems [36], agricultural system [37], and road transportation systems [38]. The eco-efficiency measure was defined as the ratio of economic output to environmental indicator variables [24,39].

### Objective, Scope, and Novel Contribution

Our objective was to develop a methodology for evaluating holistic sustainability of alternative RBZ decision management objectives (DMOs) as RBZ policy scenarios simulated separately in three 12-digit Hydrologic Unit Code (HUC12) watersheds (Back Creek, Sycamore Creek, and Greens Mill Run) within the Albemarle-Pamlico River basin (North Carolina and Virginia, USA). We evaluated the holistic sustainability of each DMO by integrating environmental indicators with economic indicators for each watershed using DEA, a widely applied statistical approach [24,26].

Building upon a previously published riparian buffer study [14], we derived three unique sets of 40, 32, and 48 DMOs, respectively, in Back Creek, Sycamore Creek, and Greens Mill watersheds. These DMOs were chosen as design strategies (the width specifications and vegetation types) needed for RBZ planners in moving from theoretical to practical decision-making. We defined five instream water quality indicator (WQI) parameters (dissolved oxygen (DO), total phosphorous (TP), total nitrogen (TN), total suspended solids as total sediment (TS), and biochemical oxygen demand (BD)) as environmental indicators and net present value (NPV) costs as economic indicators of the DMOs.

This study went beyond the previous study on RBZ-WQI parameter tradeoffs [14] and integrated the WQI parameters with the NPV costs to produce holistic sustainability scores of the DMOs by applying DEA, which to our knowledge has not been done so far. This study is intended to support integrated (holistic) riparian buffer decisions for restoring impaired waters under the 303(d) Section of the CWA.

## 2. Materials and Methods

**Figure 1.**
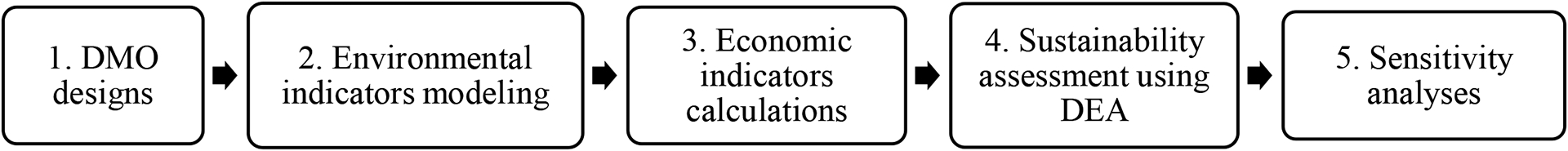
Workflow of sustainability assessment of riparian buffer zone decision management objectives (DMOs) using data envelopment analysis (DEA).

The methodology is depicted in a flow diagram (Figure 1) as described below.

### 2.1. Design of DMOs

**Figure A1.**
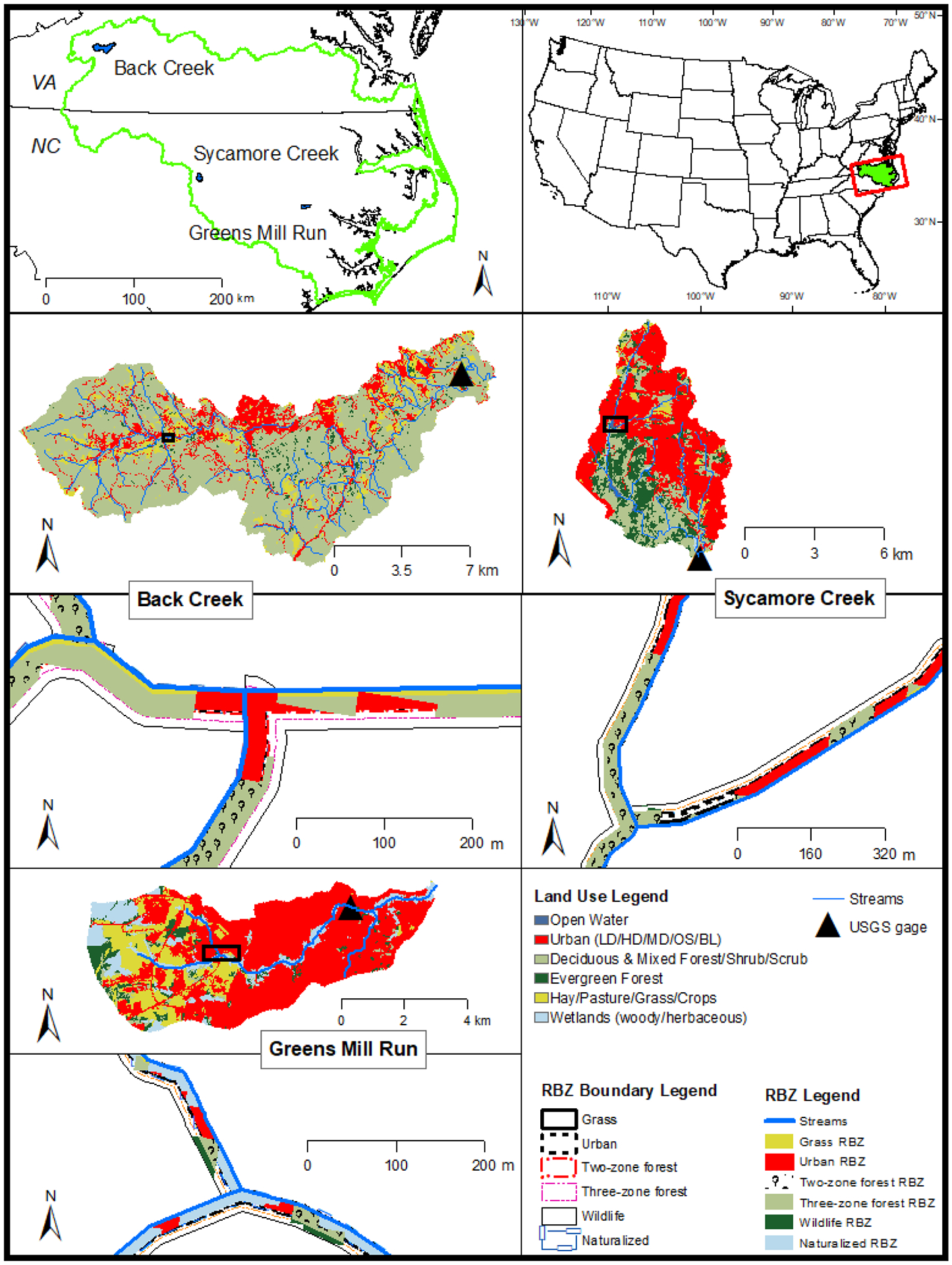
A map showing studied three watersheds, with examples of riparian buffer zone (RBZ) designs in one side of the streams in each watershed and United States Geological Survey (USGS) stream gage location (modified from Ghimire et al.) [14]). Note that the two-zone Forest RBZ and three-zone forest RBZ consisted of the same land use types (Deciduous & Mixed Forest/Shrub/Scrub), but different RBZ Legend symbols were created for clarity in the zoomed in maps of each watershed. LD = low density; HD = high density; MD = medium density; OS = open space; and BL = barren land.

We selected three HUC-12 watersheds (Back Creek, Sycamore Creek, and Greens Mill Run) situated within the Albemarle-Pamlico River basin that extends from Virginia to North Carolina (Appendix A Figure A1) and developed three conceptual unique sets of 40, 32, and 48 DMOs as RBZ policy scenarios (Tables 1 and 2). We chose these watersheds because they were previously studied by Ghimire et al. [14] and that study offered a wealth of data related to RBZ designs. These designs (Table 2) were strategically chosen from Ghimire et al. [14] by combining the baseline RBZ—width variations (25% to 200% of the baseline width) with six vegetation types (two composite vegetation types and four non-composite vegetation types) (Table 1). A single vegetation type buffer (i.e., grass, urban, naturalized, or wildlife RBZ) was termed as non-composite RBZ and a mix of two or three zones of vegetation types was termed as composite RBZ (i.e., three-zone forest or two-zone forest RBZ).

### 2.2. Environmental Indicators Modeling

We adopted the instream WQI parameters from Ghimire et al. [14] and defined environmental indicators corresponding to each DMO. Ghimire et al. [14] reported the tradeoffs between the RBZ designs and the WQI parameters (the 36-year (1983–2018) average daily simulation concentrations) using the Hydrologic and Water Quality System (HAWQS, version 1.2) [41]. HAWQS was a web-based tool developed by the U.S. EPA and Texas A&M University that employed the Soil and Water Assessment Tool (SWAT) as the core simulation engine [42]. SWAT was a widely used comprehensive watershed modeling tool for simulating the surface water and ground water conditions [42,43]. To initiate the HAWQS, the Back Creek, Sycamore Creek, and Greens Mill Run watersheds were respectively set into 12, 9, and 11 hydrologic response units (HRUs); an HRU is a lumped portion of the watershed with the similar land use types, management, and soil attributes [42]. HAWQS used the National Hydrology Dataset Plus (NHDPlus) from 2010, daily weather input data for 36 years (from 1981–2018) [41], Crop Data Layer (CDL) from 2011–2012, National Land Cover Dataset (NLCD) from 2006, and soil data retrieved from the State Soil Geographic (STATSGO) dataset [44]. The HAWQS-SWAT simulations also involved a calibration of the initial HAWQS-watershed models [27]. The calibration achieved model performance statistics of Kling–Gupta Efficiency at 0.91 for Back Creek and the Nash and Sutcliffe efficiency at 0.87 for Sycamore Creek. Refer to Ghimire et al. [14] for additional details on HAWQS-SWAT modeling.

### 2.3. Economic Indicators’ Calculations

We calculated RBZ’s long term (20-year) NPV costs as the product of unit NPV cost and the respective RBZ area (Equations (1) and (2)) using Microsoft Excel^©^ spreadsheets (Microsoft, Redmond, WA, USA):

(1) $$\text{NPV}=\frac{\sum_{i=1}^{k}A_{i}\times U_{i}}{4046.86}\;\text{for}\;\text{all}\;\text{composite}\;\text{RBZs}\;\text{with}\;i=k\;\text{number}\;\text{of}\;\text{zones}$$

(2) $$\text{NPV}=\frac{A\times U}{4046.86}\;\text{for}\;\text{all}\;\text{non}-\text{composite}\;\text{RBZs}$$

where

*NPV* = Total NPV costs ($);
*U* = Unit NPV cost ($/acre) of the baseline RBZ = the sum of the establishment, maintenance, and opportunity costs accounting for future discounting of all annual costs;
*A* = Area of an RBZ (*L* × *W*) (m^2^), where *L* is the total length of an RBZ equivalent to the total stream length within each watershed (*L* = 39,990 m, 22,940 m, and 12,070 m for the Back Creek, Greens Mill Run, and Sycamore Creek, respectively); *W* is the width of an RBZ (m) as shown in Table 2;
4046.86 = Unit conversion factor (i.e., 1 acre = 4046.86 m^2^).

When expanding Equation (1) and replacing *A*_*i*_ with *L* × *W*_*i*_, we obtained the total NPV costs equations for three-zone forest RBZ (Equation (3)) and two-zone forest RBZ (Equation (4)):

(3) $$\text{NPV}=(\frac{1}{5}U_{1}+\frac{26}{45}U_{2}+\frac{2}{9}U_{3})\times \frac{L\times W}{4046.86}$$

(4) $$\text{NPV}=(\frac{9}{35}U_{1}+\frac{26}{35}U_{2})\times \frac{L\times W}{4046.86}$$

In Equations (3) and (4), each zone of the composite RBZs varied proportional to the zone-to-composite RBZ width (*ZTW*) ratio, i.e., the ratio of the baseline zone width to the baseline composite RBZ width (Tables 1 and 2). For example, the average width of zone 1, zone 2, and zone 3 were 6.9 m, 19.8 m, and 7.6 m, respectively; and the ZTWs for zone 1, zone 2, and zone 3 of the three-zone forest RBZ were:

(5) $$ZTW_{1}=\frac{6.9}{34}=\frac{1}{5};$$

(6) $$ZTW_{2}=\frac{19.8}{34}=\frac{26}{45};$$

(7) $$ZTW_{3}=\frac{7.6}{34}=\frac{2}{9}.$$

The unit NPV costs (*U*) in Equations (3) and (4) were estimated as the sum of the establishment, maintenance, and opportunity costs per acre using the NRCS’s published values for the 2021 Fiscal Year [7]. See Table 1 for details on the NRCS Practice Case Scenarios and assumptions for unit costs for each RBZ type and the baseline RBZ widths. The maintenance costs at 10% of establishment costs were added annually for all RBZ designs in accordance with Frimpong et al. [45]. The opportunity cost due to land rental prices for the grass, urban, wildlife, three-zone forest, and two-zone forest RBZs was estimated consistent with the USDA CRP’s annual rental payment rates [40]. The opportunity cost due to cropping for all RBZs except the naturalized and urban RBZs was split between corn and soybeans, estimated consistent with the NRCS Practice Case Scenarios [7].

For all annual costs (i.e., maintenance and opportunity costs), we accounted for future discounting by estimating present value (PV) of annually recurring uniform amounts, as defined by Equation (8):

(8) $$C_{\text{PV}}=C\times \frac{{\left(1+i\right)}^{n}-1}{i\times {\left(1+i\right)}^{n}}$$

where

*C*_*PV*_ = Present value of annual cost, $;
*C* = Annual costs, $;
$\frac{{\left(1+i\right)}^{n}-1}{i\times {\left(1+i\right)}^{n}}$ = Uniform present value (UPV) factor;
*i* = Real discount rate (0.04);
*n* = Number of compounding years (RBZ lifetime = 20 years); selection of discount rate and lifetime were based on literature [17,45].

### 2.4. Evaluation of Sustainability Scores Using DEA

We used DEA to calculate the holistic sustainability scores, consistent with Ghimire and Johnston [24,26]. We defined holistic sustainability scores in terms of the classical eco-efficiency measure, defined as the ratio of economic output to environmental indicator variables [24,39]. We selected the sustainability indicators that were scientifically sound, measurable, acceptable, and relevant to the current study area, consistent with the indicator selection criteria of previously published studies [24,46,47]. For each watershed, we normalized the individual environmental indicator (i.e., WQI parameter concentration) of each DMO with respect to the mean concentration value of the indicator across all DMOs. We also normalized the economic indicator (i.e., total NPV cost) with respect to the mean NPV cost of all DMOs. The mean normalization was necessary in the preparation of DEA formulation to address data homogeneity (dimensionality) issues when integrating the environmental and economic indicators [48].

The DEA formulation began with the formal definition of eco-efficiency as the economic output divided by the linear function of environmental inputs (Equation (9)), subject to restrictions as shown in Equations (10)–(13). As such, DEA sought to minimize the environmental inputs to produce the desired economic output. To clarify Equation (9), the *n*th DMO of *N* DMOs induced *X* environmental indicators, measured by *D*_*nX*_ and each DMO had one economic indicator, *A*_*n*_:

(9) $$\text{Maximize}\;E_{n}=\frac{A_{n}}{w_{1}D_{n1}+w_{2}D_{n2}+.\ldots +w_{X}D_{nX}}(\text{for}\;\text{all}\;n=1\;\text{to}\;N)$$

subject to

(10) $$\frac{A_{1}}{w_{1}D_{11}+w_{2}D_{12}+.\ldots +w_{X}D_{1X}}\leq 1$$

(11) $$\frac{A_{2}}{w_{1}D_{21}+w_{2}D_{22}+.\ldots +w_{X}D_{2X}}\leq 1$$

(12) $$\frac{A_{N}}{w_{1}D_{N1}+w_{2}D_{N2}+.\ldots +w_{X}D_{NX}}\leq 1$$

(13) $$w_{1},w_{2},\ldots w_{X}\geq 0$$

where

*E* = holistic sustainability score;
*A* = economic indicator;
*D* = environmental indicator;
*w*_*i*_ = model weights estimated by DEA optimization, *i* ranged from 1 to *X*, the number of environmental indicators (in this study, *X* = 5).

Equation (9) and the restrictions (Equations (10)–(12)) were nonlinear functions and therefore transformed to linear form by determining the inverse functions (Equation (14)) with the variables defined in the opposite direction (Equations (15)–(17)). We solved these mathematical models and obtained the eco-efficiency measure as a sustainability score by taking an inverse of the solution (Equation (14)):

(14) $$\text{Minimize}\;E^{-1}{}_{n}=\frac{1}{A_{n}}\left(w_{1}D_{n1}+w_{2}D_{n2}\ldots +w_{X}D_{nX}\right)$$

subject to

(15) $$\frac{w_{1}D_{11}}{A_{1}}+\frac{w_{2}D_{12}}{A_{1}}+.\ldots +\frac{w_{X}D_{1X}}{A_{1}}\geq 1;$$

(16) $$\frac{w_{1}D_{21}}{A_{2}}+\frac{w_{2}D_{22}}{A_{2}}+.\ldots +\frac{w_{X}D_{2X}}{A_{2}}\geq 1;$$

(17) $$\frac{w_{1}D_{n1}}{A_{n}}+\frac{w_{2}D_{n2}}{A_{n}}+.\ldots +\frac{w_{X}D_{nX}}{A_{n}}\geq 1;$$

(18) $$w_{1},w_{2},\ldots w_{X}\geq 0.$$

Furthermore, the classical DEA was improved by imposing an additional weighting scheme of equal weights (Equation (19)) assuming each environmental indicator received equal importance, consistent with [24] what was later applied by Ghimire and Johnston [26] for evaluating sustainability of alternative agricultural systems comprising crop types and irrigation practices:

(19) $$w_{1}=w_{2}=\ldots ..=w_{X}$$

In this case, *X* = 5 and weights corresponded as: *w*_1_ = DO^−1^; *w*_2_ = TP; *w*_3_ = TN; *w*_4_ = TS; and *w*_5_ = BD. Note that the mean normalized DO values were inverted (DO^−1^) to be consistent with other environmental indicators that were to be minimized for optimal performance of a DMO, meaning that a decrease in the value of each of these indicators was considered advantageous.

To initiate the DEA, a random number between 0 to 1 was generated as an initial value of each *w*_*i*_ of each DMO that was then optimized by DEA. The improved DEA formulation was solved for each DMO incorporating the equal weighting scheme in addition to the classical DEA constraints, and a holistic sustainability score was estimated by calculating the inverse of the DEA-optimized scores. Microsoft Excel^©^ was used for all calculations. An example of DEA formulation is provided in the Appendix A.

Finally, we ranked the holistic sustainability scores of the DMOs in each watershed to assess the DMO sustainability tradeoffs and to identify the most- and least-sustainable DMOs.

### 2.5. Sensitivity Analyses

To determine how changes in key input values of assumptions related to NPV cost assessments and the DEA impacted the findings, we performed sensitivity analyses of NPV costs and weighting schemes separately:

Sensitivity of NPV costs variation: As shown in Equation (1) through 4, the total NPV costs was the function of the unit NPV costs (*U*), stream length (*L* ), and RBZ width (*W*). To determine how changes in input values of assumptions related to lifetime and discount rates affect *NPV*, we tested sensitivity of the RBZ lifetime (5 years to 80 years) and three different discount rates (0.03, 0.04, 0.05) to *U*. The RBZ lifetimes and the discount rates were chosen consistent with literature values [45,49].

Sensitivity of weighting schemes: To determine how changes in input values of assumptions in DEA Equations (15)–(18) (i.e., equal versus unequal weights) affect the holistic sustainability score (*E*), we imposed an unequal weighting scheme (Equation (20)) that was similar to the National Institute of Standards and Technology (NIST) panel weighting scheme [24,50] and previously examined by Ghimire and Johnston [24]:

(20) $$w_{4}\geq w_{3}\geq w_{5}\geq w_{2}\geq w_{1}$$

Note that Ghimire and Johnston [24] addressed the sensitivity and subjectivity requirements of sustainability analyses by assessing the performance of 10 weighting schemes including classical DEA, equal weights, Eco-Indicator 99 [51], Sustainable Society Index scheme [52], NIST stakeholder panel scheme [50], and five derived impact threshold schemes. They recommended equal weights and the threshold schemes to overcome the non-uniqueness problem in sustainability analyses.

No further sensitivity of environmental indicators was conducted because these data were adopted from the previously calibrated HAWQS-SWAT modeling [14].

## 3. Results

### 3.1. RBZ DMO Designs

We evaluated the holistic sustainability of each DMO for each watershed (described in Tables 1 and 2) separately and shed light on the sustainability of the RBZ policy scenarios. The DMOs varied by RBZ widths and vegetation (grass, urban, naturalized, wildlife, three-zone forest, and two-zone forest).

### 3.2. Environmental Indicators

**Figure 2.**
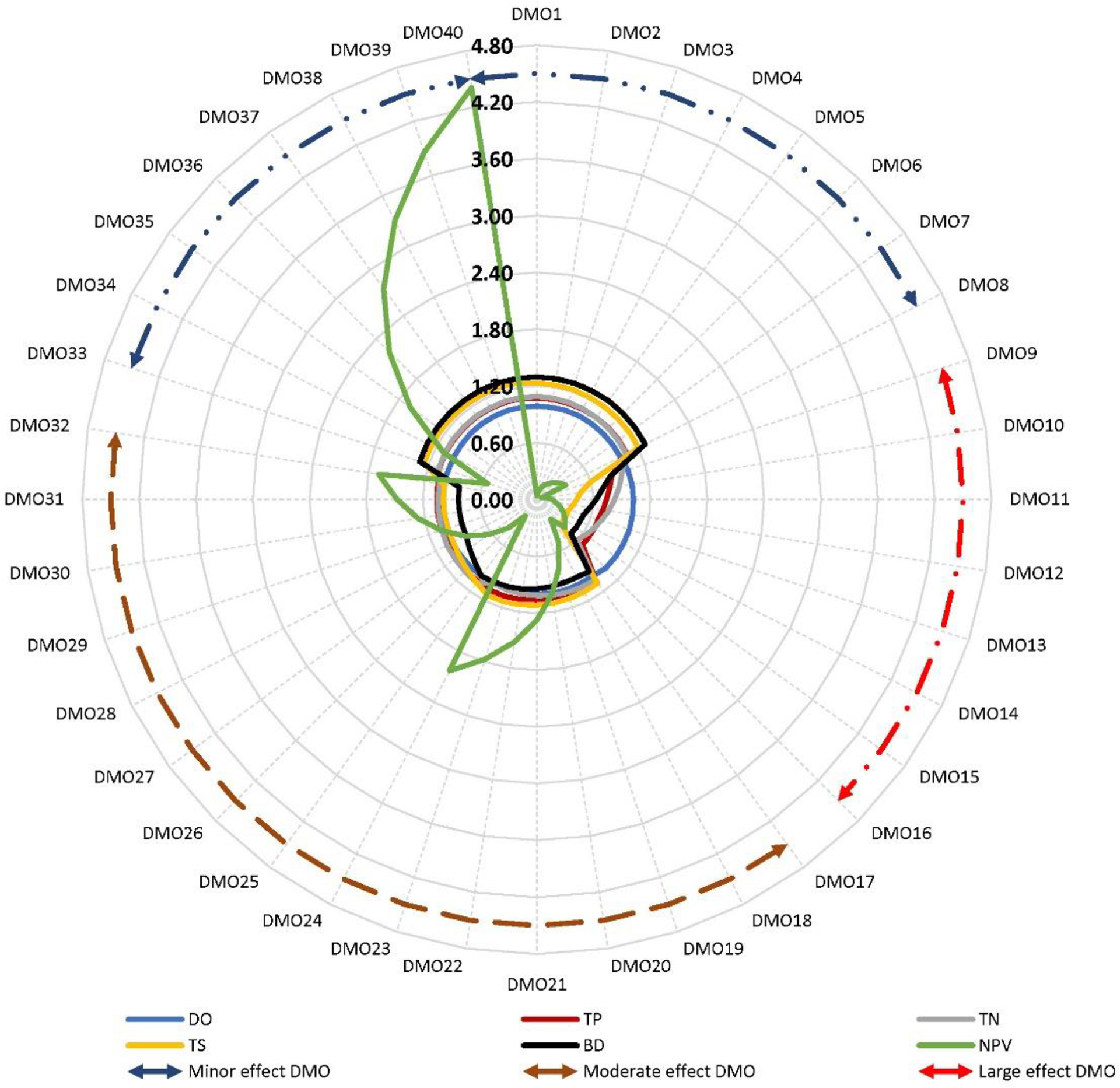
Mean normalized environmental indicators (DO, TP, TN, TS, and BD) and economic indicators (NPV costs) of the 40 RBZ DMOs in Back Creek. Circular arrows indicate the DMOs that correspond to the minor effects (dark blue dotted outline), moderate effects (dark red dotted outline), and large effects (red dotted outline). Refer to Table 2 for the DMO descriptions.

**Figure 3.**
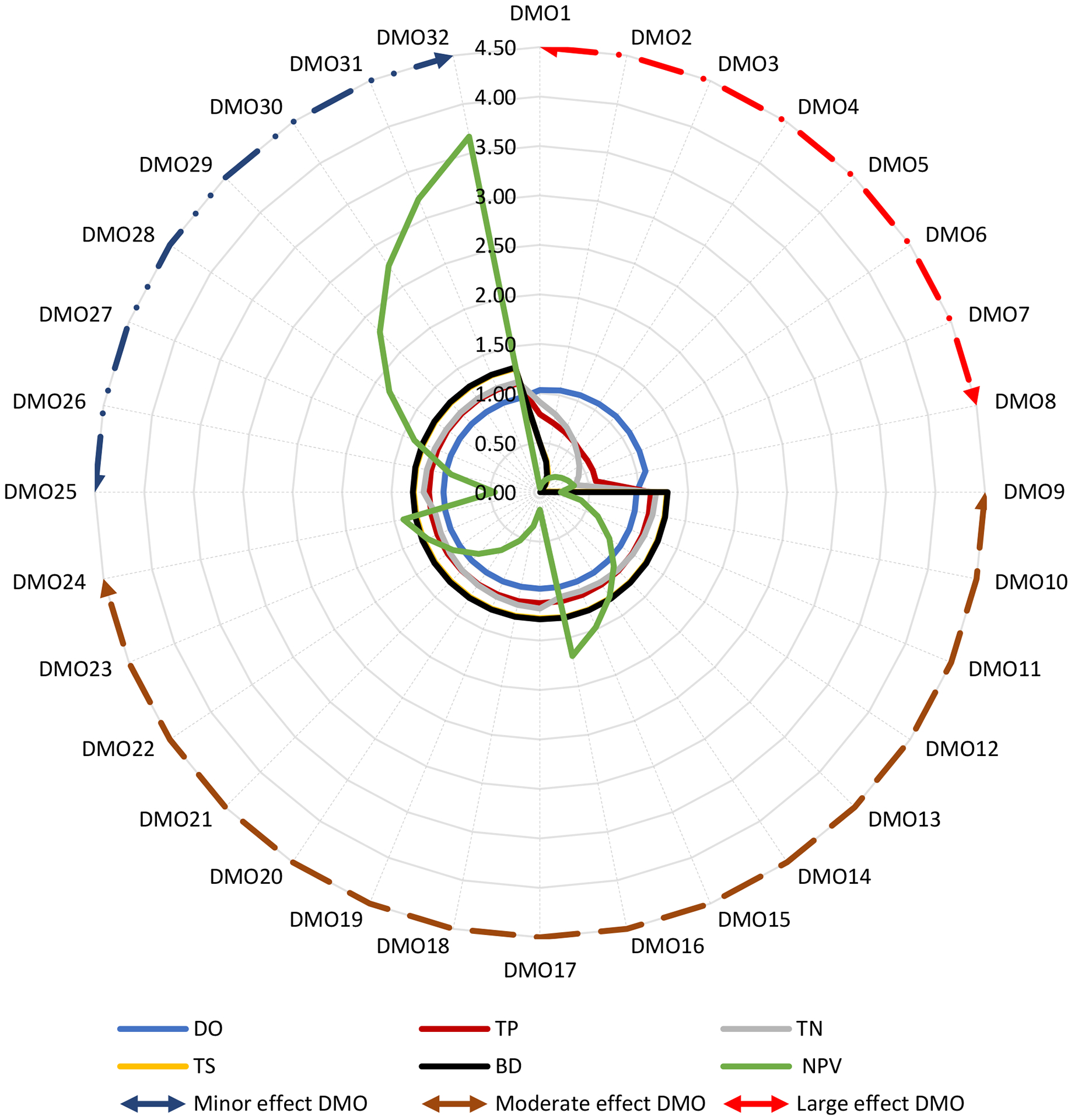
Mean normalized environmental indicators (DO, TP, TN, TS, and BD) and economic indicators (NPV costs) of the 32 RBZ DMOs in Sycamore Creek. Circular arrows indicate the DMOs that correspond to the minor effects (dark blue dotted outline), moderate effects (dark red dotted outline), and large effects (red dotted outline). Refer to Table 2 for the DMO descriptions.

**Figure 4.**
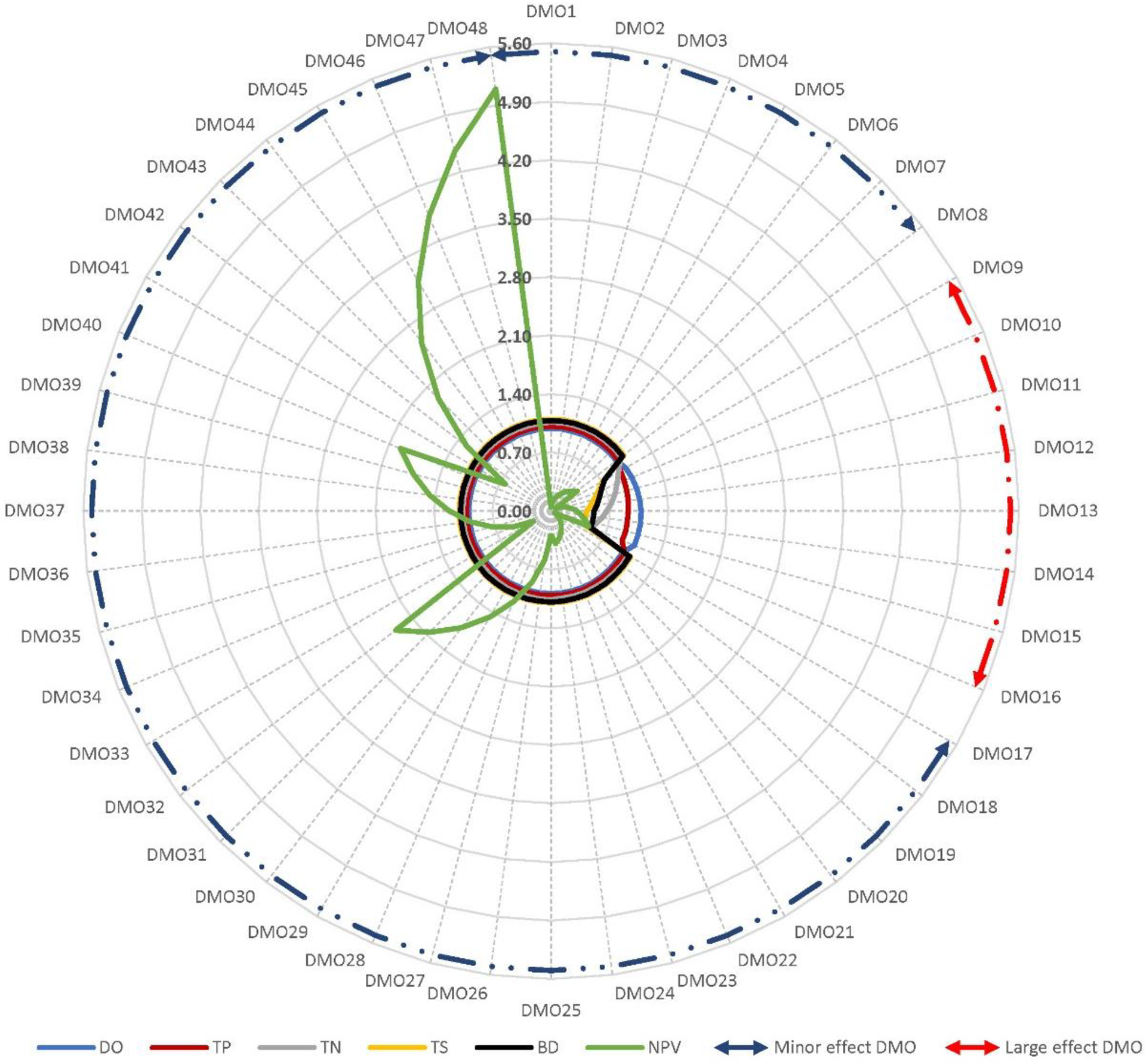
Mean normalized environmental indicators (DO, TP, TN, TS, and BD) and economic indicators (NPV costs) of the 48 RBZ DMOs in Greens Mill Run. Circular arrows indicate the DMOs that correspond to the minor effects (dark blue dotted outline) and large effects (red dotted outline). Refer to Table 2 for the DMO descriptions.

The comparison of the mean normalized environmental indicators of the DMOs showed watershed-specific variations (Figures 2–4). In Back Creek, these indicators showed relatively minor effects (≤5%), moderate effects (5% to 20%), and large effects (>20%). As shown in Figure 2, all environmental indicators of DMO1 through DMO8 (i.e., grass RBZ—width variations) and DMO33 through DMO40 (i.e., wildlife RBZ—width variations) showed relatively minor effects (≤5%)—from no change in DO to up to a 5% change in TS (120% due to DMO8 to 125% due to DMO33).

All indicators except the DO of the DMO17 through DMO32 (i.e., composite RBZs, the three-zone forest RBZ—width and two-zone forest RBZ—width variations) showed moderate effects (5% to 20%)—from 15% change in TS (99% due to DMO32 to 114% due to DMO24) to 17% change in BD (83% due to DMO32 to 100% due to DMO 25). Furthermore, all except the DO of the DMO9 through DMO16 (i.e., urban RBZ width variations) showed large effects (>20%)—from 25% change in TS (35% due to DMO16 to 60% due to DMO9) to 35% change in TN (60% due to DMO16 to 95% due to DMO9).

In Sycamore Creek, all indicators of DMO25 through DMO32 (i.e., wildlife RBZ—width variations) showed relatively minor effects (_5%)—from no change in DO to 4% change in TN (114% to 118%); only TN of DMO9 through DMO24 (i.e., three-zone forest RBZ and two-zone forest RBZ variations) showed moderate effects (108% to 118%); and all except the DO of DMO1 through DMO8 (i.e., urban RBZ width variations) showed large effects—from a 21% change in TP (58% to 79%) to a 54% change in TN (37% to 91%) (Figure 3).

In case of Greens Mill, the normalized environmental indicators showed minor effects (≤5%) of all DMOs except the urban RBZ width variations (i.e., DMO8 through DMO16), of which all but the DO showed large effects, from 22% change in BD (51% to 73%) to 41% TN (52% to 93%) (Figure 4).

### 3.3. Economic Indicators

**Figure A2.**
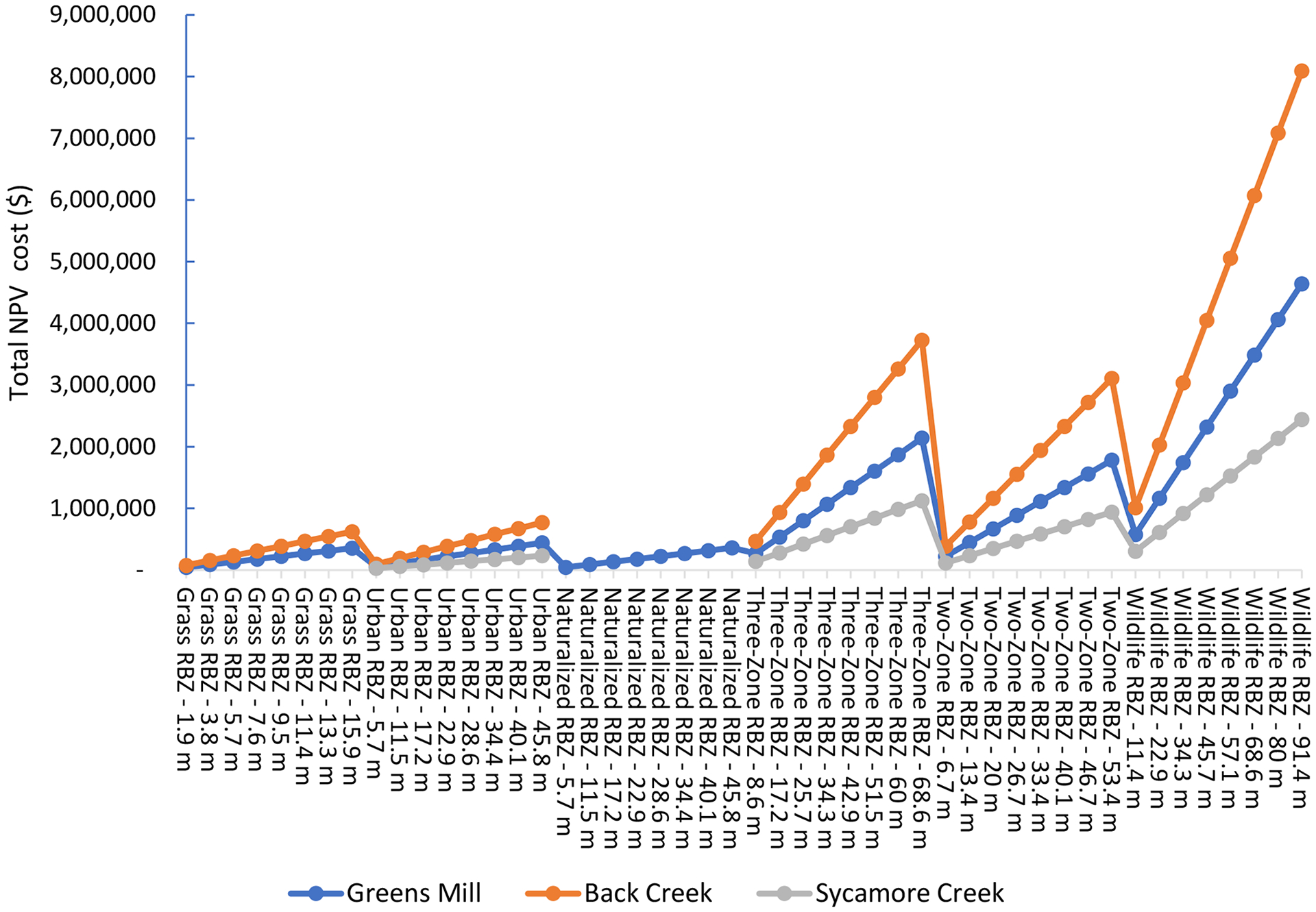
Total NPV costs of RBZ DMOs in three watersheds.

**Figure A3.**
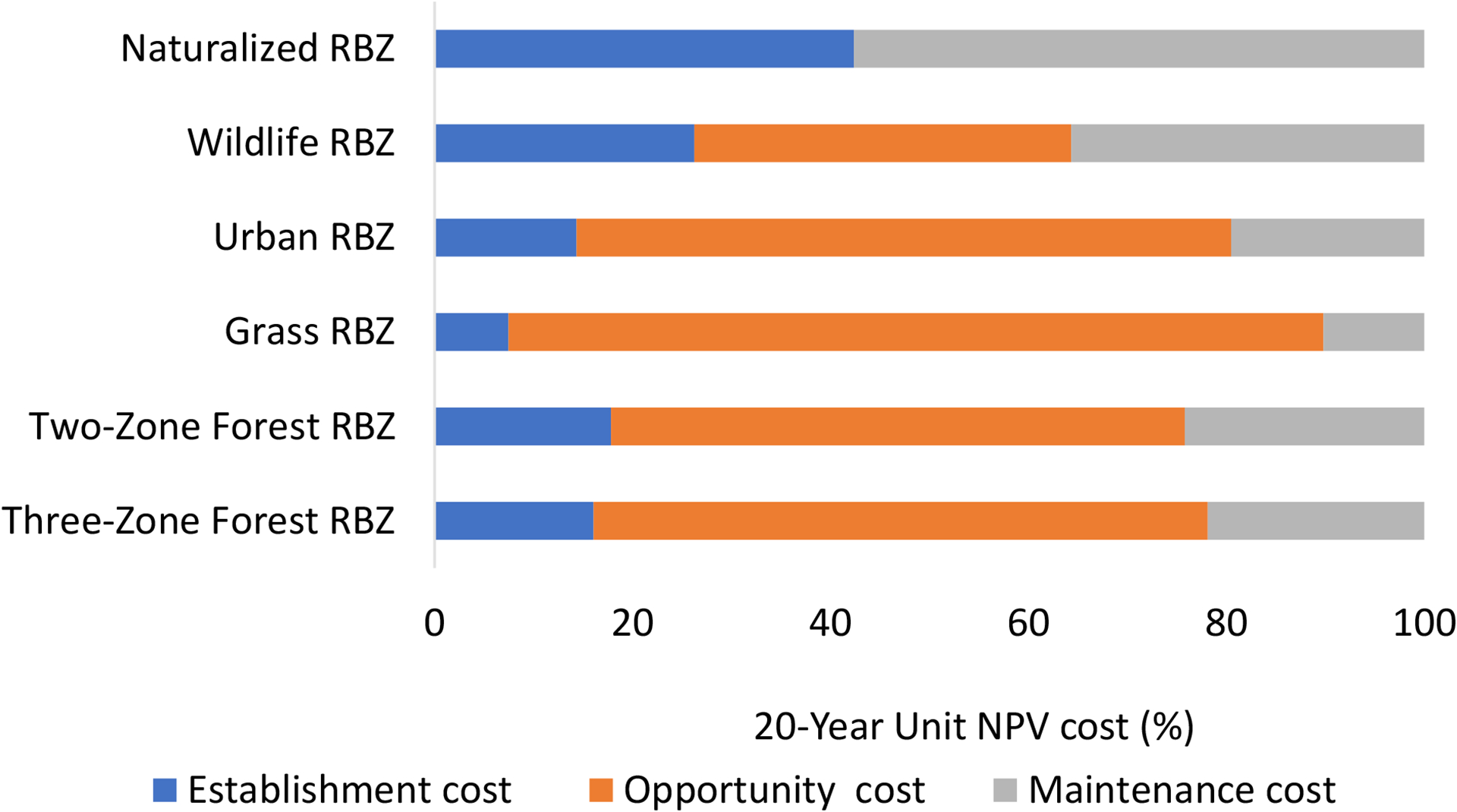
Percentage (%) contribution of establishment cost, opportunity cost, and maintenance cost to unit NPV cost.

The mean normalized economic indicators (NPV costs) of each of the DMOs varied by the RBZ’s width, length, and unit NPV cost—from 4% (grass RBZ—1.9 m) to 400% (wildlife RBZ—91.4 m) in Back Creek, 4% (urban RBZ—5.7 m) to 400% (wildlife RBZ—91.4 m) in Sycamore Creek, and 5% (grass RBZ—1.9 m) to 500% (wildlife RBZ—91.4 m) in Greens Mill Run (Figures 2–4). The unit 20-y NPV costs of the RBZs varied from $3430/hectare (naturalized RBZ) to $22,146/hectare (wildlife RBZ), and the estimated annualized NPV costs ranged from $252/hectare (naturalized RBZ) to $1628/hectare (wildlife RBZ) (Tables 3 and A4). Note that the NPV cost was related to the RBZ’s width, length, and the unit NPV cost that was the sum of the establishment, maintenance, and opportunity costs accounting for future discounting of all annual costs (see Equations (3), (4), and (8)). The NPV costs of the DMOs were highest for Back Creek watershed, consistent with the longest RBZ lengths (Appendix A Figure A2). The opportunity costs dominated the unit NPV costs of the four baseline RBZs, including two-zone forest (58%), three-zone forest (62%), urban (66%), and grass RBZ (82%), due primarily to the annual income of cropland and land rental (Table 3 and Appendix A Figure A3). The opportunity cost also influenced wildlife RBZ costs (38%) but had no influence (0%) on the naturalized RBZ costs.

### 3.4. Holistic Sustainability

**Figure 5.**
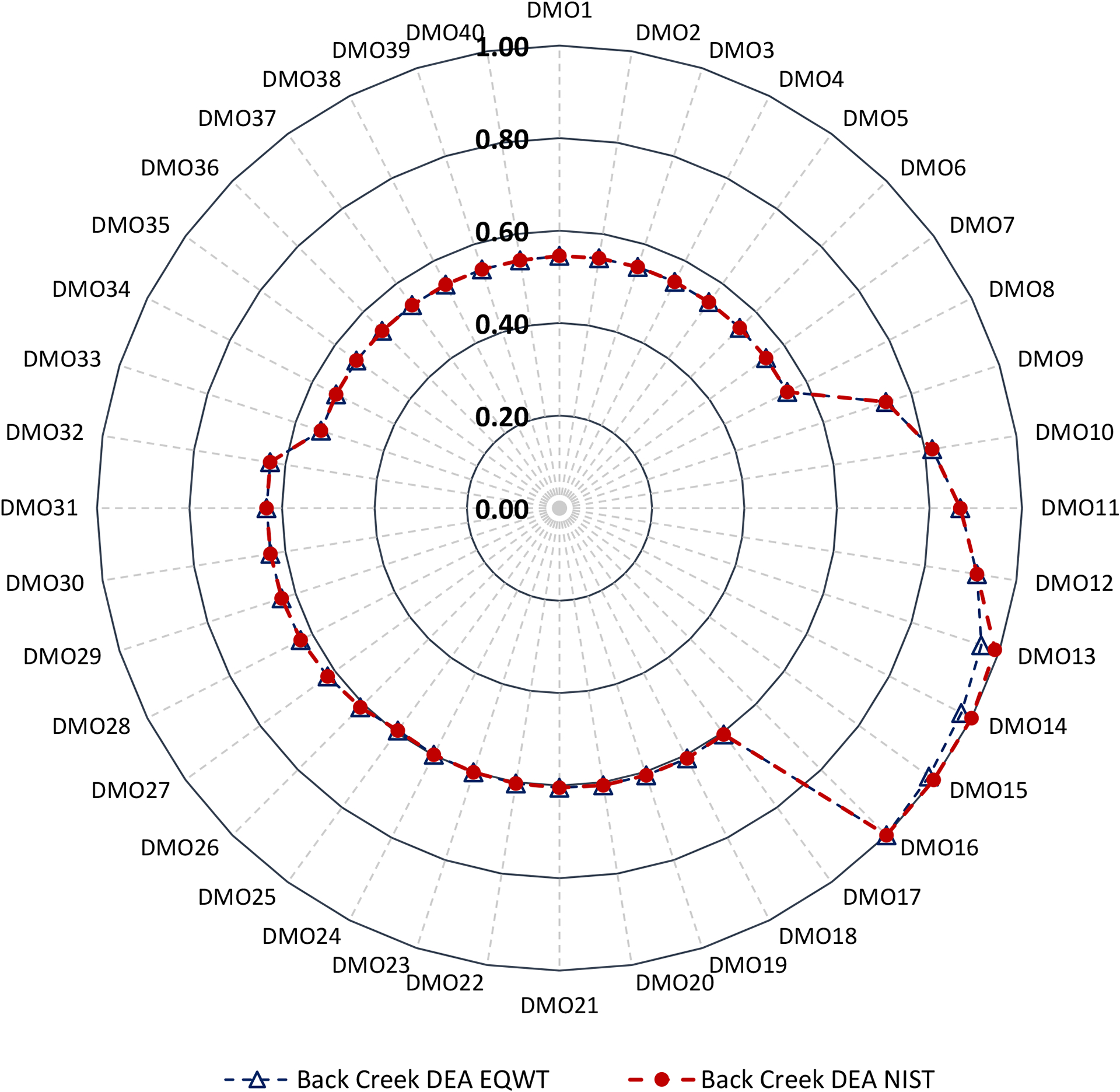
Back Creek RBZ DMO sustainability scores using the equal weight (EQWT) scheme and unequal weight scheme (similar to the National Institute of Standards and Technology (NIST) stakeholder panel scheme). Refer to Table 2 for the DMO descriptions.

**Figure 6.**
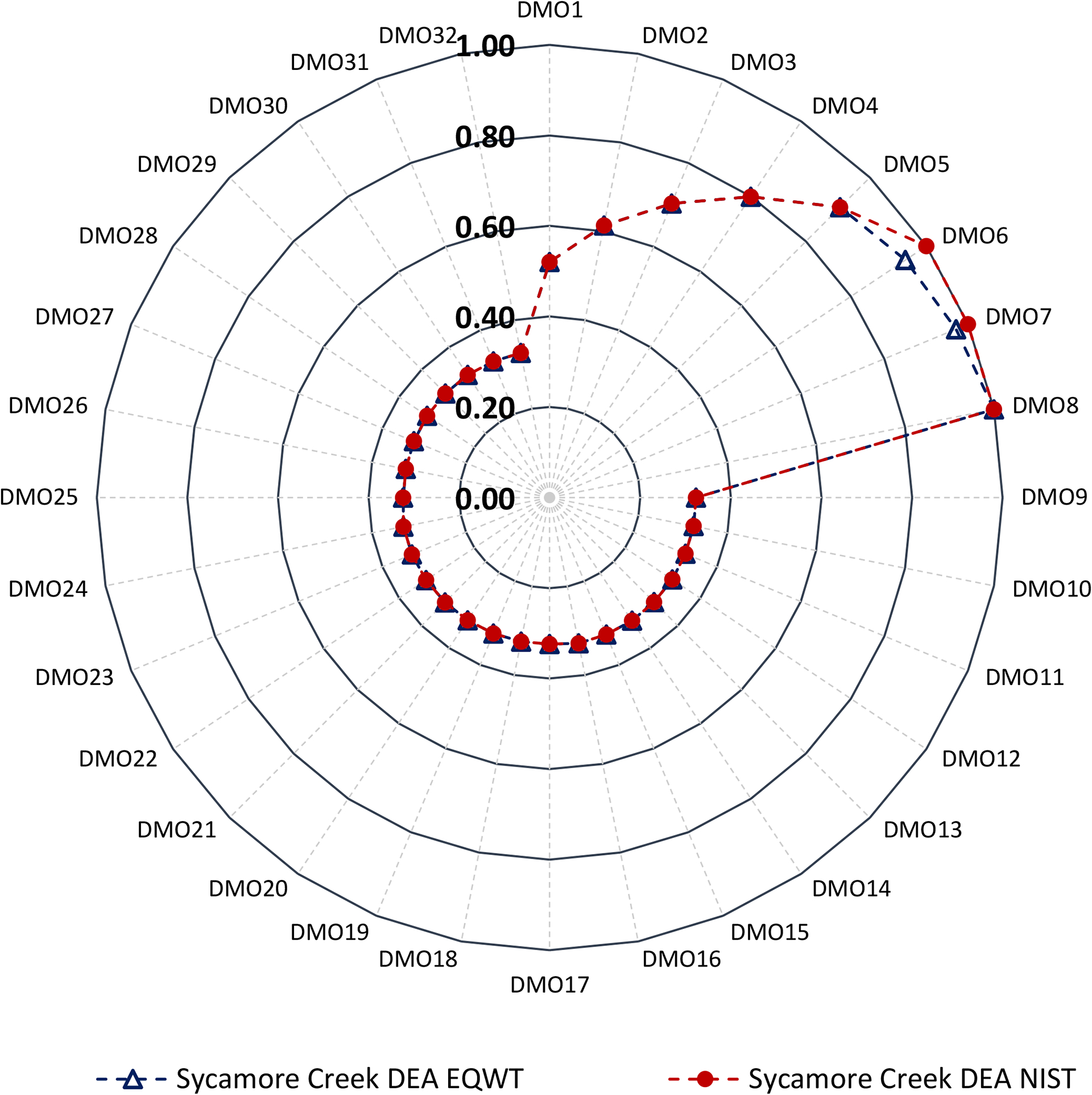
Sycamore Creek RBZ DMO sustainability scores using the equal weight (EQWT) scheme and unequal weight scheme (similar to the National Institute of Standards and Technology (NIST) stakeholder panel scheme). Refer to Table 2 for the DMO descriptions.

**Figure 7.**
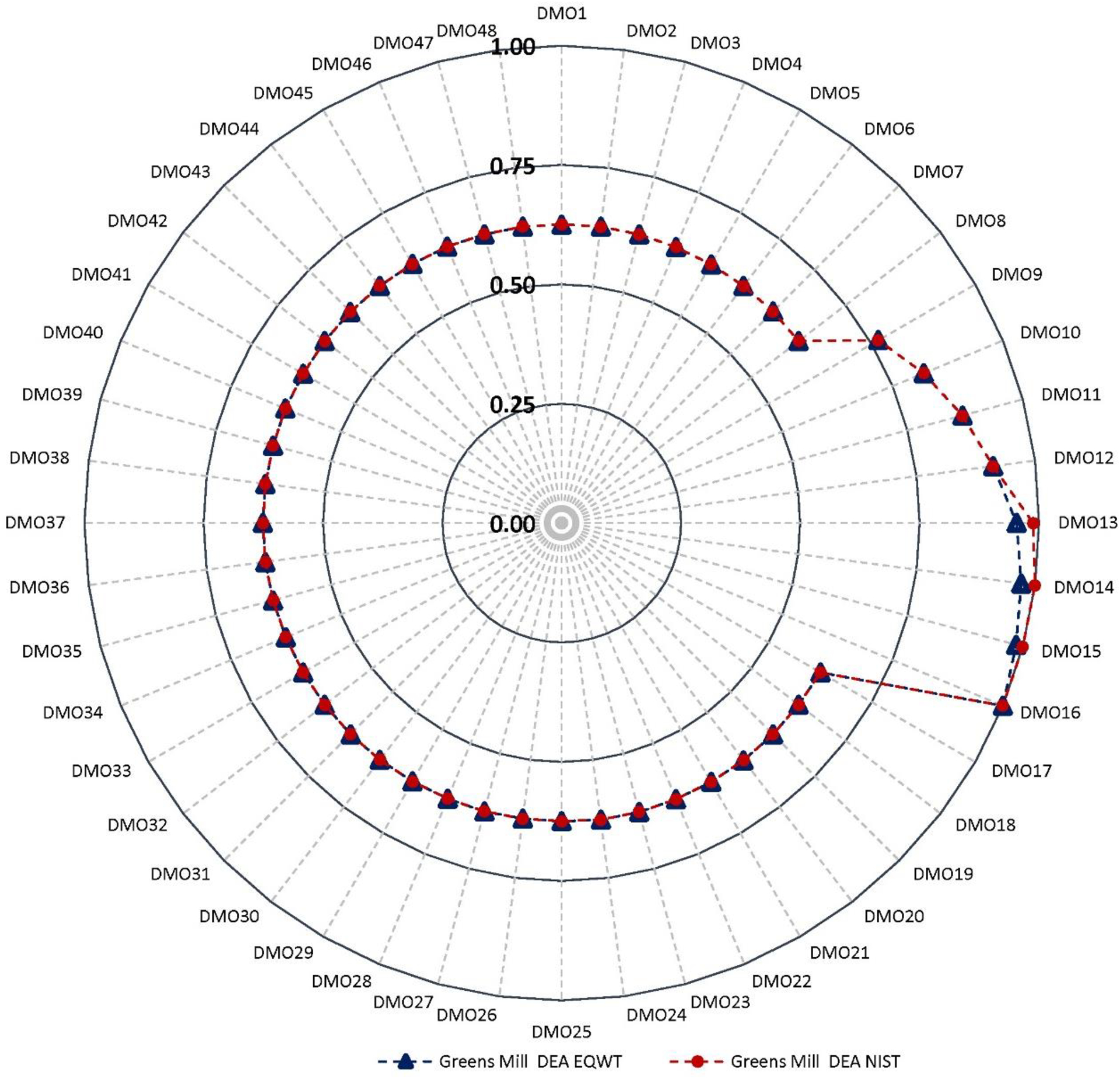
Greens Mill Run RBZ DMO sustainability scores using the equal weight (EQWT) scheme and unequal weight scheme (similar to the National Institute of Standards and Technology (NIST) stakeholder panel scheme). Refer to Table 2 for the DMO descriptions.

To evaluate the holistic sustainability of each DMO, we integrated the environmental indicators with economic indicators using DEA. The holistic (integrated) sustainability scores of the three sets of DMOs for each watershed are depicted in Figures 5–7. The sustainability scores ranged from 0.54 (wildlife RBZ—11.4 m, DMO33) to 1.00 (urban RBZ—45.8 m, DMO16) for Back Creek, 0.32 (three-zone forest RBZ—8.6 m) to 1.00 (urban RBZ—45.8 m) for Sycamore Creek, and 0.62 (three-zone forest RBZ—8.6 m) to 1.00 (urban RBZ—45.8 m) for Greens Mill, revealing the urban RBZ—45.8 m as the most sustainable DMO across all watersheds. Note that the sustainability scores were evaluated in relationship to each other (i.e., DMOs) that were optimized within each watershed separately.

The ranking of the sustainability scores placed all eight urban RBZ width variations at the top for each watershed, with sustainability scores of urban RBZ—5.7 m to 45.8 m ranging from 0.74 to 1.00 in Back Creek, 0.52 to 1.00 in Sycamore Creek, and 0.77 to 1.00 in Greens Mill Run. However, the least sustainable DMOs varied by watershed; all the eight wildlife RBZ DMOs (wildlife RBZ—11.4 m to 91.4 m) were found at the bottom, with an equivalent sustainability score of 0.54 in Back Creek; the three-zone forest, two-zone forest, and the wildlife RBZs were found at the bottom, with an equivalent sustainability score of 0.32 in Sycamore Creek; and the three-zone forest and two-zone forest RBZs were found at bottom, with an equivalent sustainability score of 0.62 in Greens Mill.

### 3.5. Cost Sensitivity

**Figure A6.**
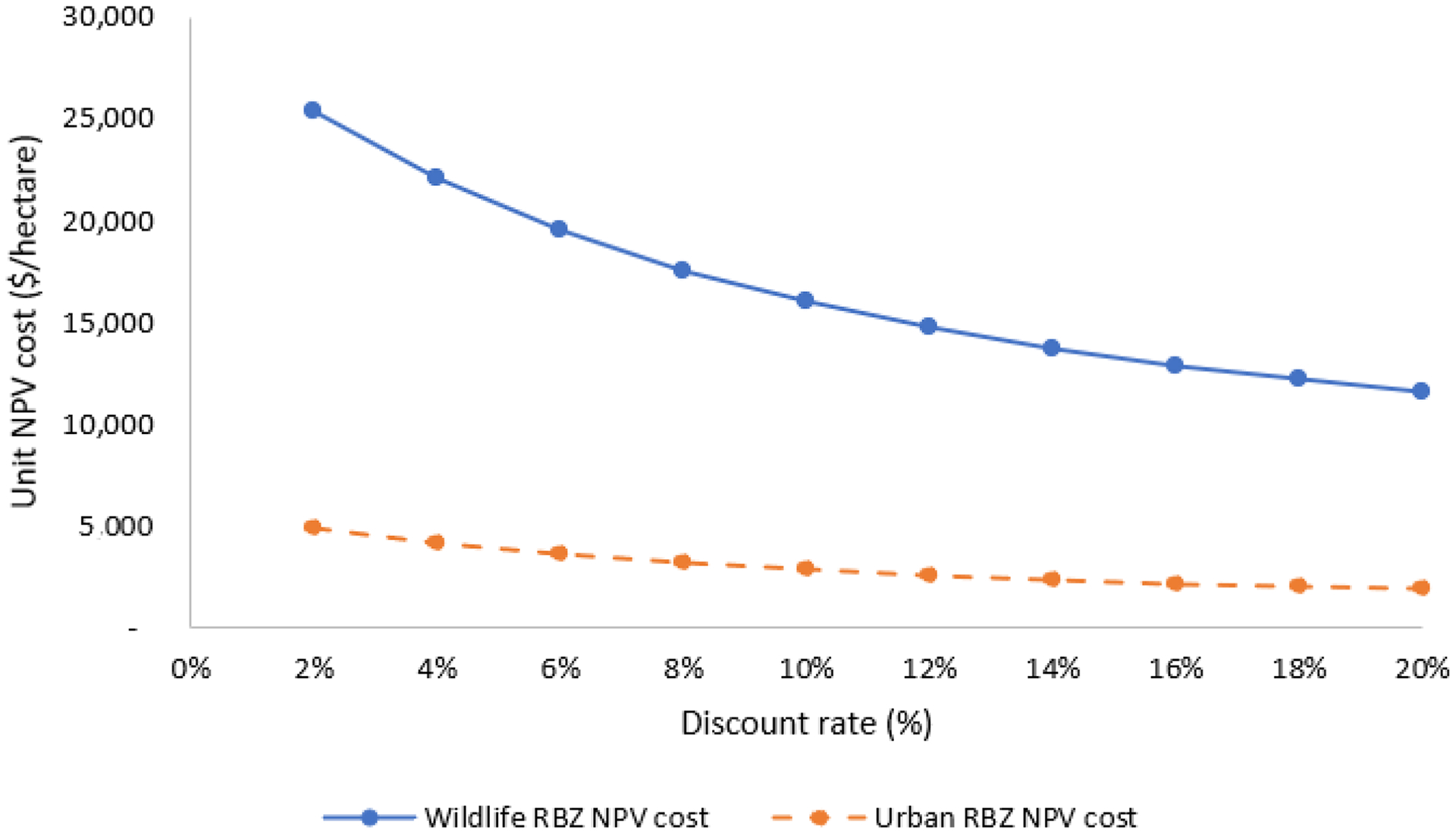
Sensitivity of the discount rates to the unit NPV costs.

The opportunity costs were found to be most influential to the NPV costs for five of the six baseline RBZs primarily due to the annual income as functions of the RBZ lifetime and the discount rate. Furthermore, the sensitivity analyses of RBZ lifetime and discount rate to unit NPV costs showed that the costs increased logarithmically over the lifetime (Appendix A Figure A6). For the urban RBZ lifetime variation from 5 years to 80 years, the unit NPV costs with a 3% discount rate ranged from $1821/hectare to $8624/hectare; the NPV costs with a 5% discount rate were lower, ranging from $1754/hectare to $5808/hectare (Appendix A Figure A6). For the wildlife RBZ lifetime variation, the unit NPV costs with a 3% discount rate ranged from $11,316/hectare to $ 42,109/hectare, which were higher than the urban RBZs (Appendix A Figure A6). The opportunity costs of grass, three-zone forest, and two-zone forest RBZs were the same as the wildlife RBZ and thus follow similar cost variation patterns.

### 3.6. Weighting Scheme Sensitivity

The NIST weighting scheme produced an optimal score of sustainability of 1.00 for three DMOs of urban RBZs (urban RBZ—34.4 m, 40.1 m, and 45.8 m) for all watersheds (Figures 5–7). However, the EQWT scheme addressed this uniformity having values consistent with the previous study [24]. Overall, the urban RBZ—34.4 m—was found to be the most sustainable DMO in each watershed.

## 4. Discussion

The study suggested watershed-specific yet generalizable sustainability implications of RBZ policy scenarios that were simulated in the Back Creek, Sycamore Creek, and Greens Mill Run watersheds.

The mean normalization of the environmental indicators not only addressed the limitations of data size (non-homogeneity) in DEA [24,48] but also furthered the understanding of RBZ policy scenarios consistent with the previously reported RBZ design—WQI parameter tradeoffs [14]. For example, in the Back Creek watershed, the indicators showed minor, moderate, and large effects (Figure 2) suggesting three levels of potential improvements in WQI parameters corresponding to the grass or wildlife RBZ, the composite RBZs (i.e., two-zone forest and three-zone forest), and the urban RBZ implementations, respectively. The previous sensitivity analyses reported optimal width for each RBZ design in each watershed [14]. They reported urban RBZ width variation to be most sensitive with the largest effects variations across all watersheds, with TS from 61–96%, TN from 34–55%, TP from 9–48%, and BD from 53–99% compared to no-RBZ conditions. Moreover, they found the other RBZ width variations to be less sensitive than urban RBZ, with minimal effects (<5%) of wildlife RBZ and grass RBZ widths on all WQI parameters in all watersheds and moderate effects of composite RBZ widths in Back Creek.

However, the normalized economic indicators varied from 4% (grass RBZ—1.9 m) in Back Creek to 500% (wildlife RBZ—91.4 m) in Greens Mill Run, suggesting grass RBZ policy scenarios as the least expensive. The economic indicators (NPV costs) of each of the six baseline RBZ types varied by watershed according to the RBZ’s width, length, and the unit NPV costs (Equations (3) and (4)). The unit NPV costs of the baseline RBZs ranged from $3430/hectare or $1388/acre (naturalized RBZ) to $22,146/hectare or $8962/acre (wildlife RBZ) for each watershed (Table 3). To compare the estimated NPV costs with past studies, we estimated the RBZ’s annualized NPV costs by aggregating the establishment costs with the annually occurring maintenance costs and opportunity costs over the expected RBZ life of 20 years, consistent with Roberts et al. [19]. The estimated annualized NPV costs of the six-baseline RBZs, grass, urban, naturalized, two-zone forest, three zone forest, and wildlife RBZ, were $754/hectare, $309/hectare, $252/hectare, $1070/hectare, $1001/hectare, and $ 1628/hectare, respectively. These cost estimates were comparable to past studies that suggested a range of RBZ costs. For example, Roberts et al. [19] estimated annual costs ranging from $128 to $867/hectare for buffer strips in a watershed in Tennessee (USA), Tyndall and Bowman [53] estimated the RBZ annual costs ranging from $576 to $890/hectare for a watershed in Iowa (USA), and LeDoux [54] reported annual protection costs of forested RBZ in terms of opportunity costs ranging from $378 to $1653/hectare in eastern hardwood forests.

While the understanding of these economic and environmental indicators was important for assessing the RBZ policies considering individual indicator tradeoffs, a single array of integrated sustainability scores served as a practical decision-making tool for RBZ planners, especially due to the multiple preferences and levels of tradeoffs associated with individual indicators. The implementation of a sustainable RBZ in a watershed can realize economic value in terms of the mean normalized NPV costs and improve the environmental (water quality) impacts. These results can be supportive for making informed RBZ decisions to protect and restore stream water quality under Section 303(d) of the Clean Water Act (CWA) and help meet the goals of the USDA’s Conservation Reserve Program (CRP). An RBZ planner can use the holistic scores (as reported in Figures 5–7) to select a specific DMO as the most sustainable RBZ policy scenario, or alternatively choose a near-optimal DMO (an equivalent ranking DMO) as an alternative RBZ policy. For example, in Back Creek, the scores of the most sustainable urban RBZ—45.8 m (1.00) were equivalent to the second- and third ranking urban RBZs—40.1 m (0.98) and urban RBZ—34.4 m (0.98), which involved narrower width but offered the same function. The findings of urban RBZ policy scenarios as most sustainable among others underscore the need of integrated urban buffer planning policies to sustain urban ecosystems and to promote water security in the face of the currently growing global urbanization that is projected to double by 2050 [55,56]. The findings reported may be used to inform stakeholders, homeowners, and developers on the importance of urban RBZ and greenways in protecting streams’ water quality.

The sustainability score also indicated potential room for improvement in one or more indicators of interest that may be controlled using alternate RBZs with innovative technologies. For example, the sustainability score (0.40) of the least sustainable DMO33 (wildlife RBZ—11.4 m) in Back Creek, or elsewhere, could be improved by targeting alternate timber logging technologies with relatively smaller opportunity costs for the RBZ maintenance. In fact, past studies suggested that streamside standing timbers represented a significant portion of opportunity costs that varied by tree species and the logging technology needed for RBZ maintenance [54,57].

We advise caution in interpreting the sustainability scores within a watershed as the sustainability scores were optimized in relationship to DMOs and the indicators that were optimized within the watershed separately. In addition, the selection of sustainability indicators vary with the goal and the scope of research; for example, whereas the indicators of sustainable watershed management may range from water quality to life cycle global warming impacts [24,46], the indicators of sustainable development goals may incorporate an array of the economic, environmental, and social pillars [24,46,58]. Hence, the holistic sustainability assessment is subjective due to the involved multidisciplinary indicators and subjectivity of the stakeholders’ views on the weights of the pillars of sustainability.

Following up the previously published RBZ—stream water quality modeling [14], the current study involved a second step towards holistic watershed management through a cross-disciplinary approach of sustainability. In the long-term, additional environmental indicators such as biologic carbon sequestration (atmospheric CO_2_ in vegetation and woody products), global warming potential, and eutrophication potential could be easily incorporated into current methods. Life cycle assessment (LCA) can be used to calculate such indicators [24,26]. Other social indicators resulting from RBZ implementations, such as improvement or depletion in fisheries and improvements in physical and emotional well-being due to increased living standards, employment, and other benefits, could also be systematically assessed (e.g., contingent valuation involving willingness to pay for avoided- or to be paid for accepted environmental impacts) and incorporated into future methods.

The current study demonstrated an approach to address holistic sustainability of streamside RBZ policy scenarios focusing on water quality and NPV costs as environmental and economic indicators. A sustainability assessment of RBZs applied to other waterbodies such as wetlands, ponds, and other sources of water were beyond the scope of the current study; however, the presented methods can be applied to assess such scenarios, e.g., buffered wetlands [59], by obtaining watershed-specific information, plant allocation [60], tree species, and logging technology, and considering government programs such as the Wetlands Reserve Program (WRP) that offer preferred cost sharing for landowners [61,62].

## 5. Conclusions

We demonstrated a methodology to evaluate holistic sustainability of three unique sets of 40, 32, and 48 RBZ policy scenarios (the DMOs) in three HUC-12 watersheds (Back Creek, Sycamore Creek, and Greens Mill Run) located in the southeastern U.S. The three sets of the DMOs comprised the variation in RBZ widths (25% to 200% baseline width) and the six vegetation types (grass, urban, naturalized, wildlife, three-zone forest, and two-zone forest). The baseline grass, urban, two-zone forest, three-zone forest, wildlife, and naturalized RBZs were 8, 23, 27, 34, 46, and 23 m wide, respectively. We adopted the WQI parameter concentrations (DO, TP, TN, TS, and BD) previously published by Ghimire et al. [14] as environmental indicators and developed methods to calculate NPV costs utilizing the establishment, maintenance, and the opportunity costs based upon the statewide billing practices for FY2021 set by the NRCS. Using a widely applied statistical tool called DEA, we integrated these environmental and economic indicators to evaluate the DMOs’ holistic sustainability scores uniquely within each watershed. The study is intended to support practical decision making related to RBZ policy scenarios using explicit sustainability score tradeoffs. We summarize key study implications below:

- The NPV costs varied by DMOs across the watersheds, ranging from 4% (grass RBZ—1.9 m) in Back Creek to 500% (wildlife RBZ—91.4 m) in Greens Mill compared to the mean total NPV costs—due primarily to the greater width and the opportunity costs of the corresponding RBZ.
- Cost sensitivity analyses showed that the opportunity costs dominated the unit NPV costs of the baseline RBZs from 58% (two-zone forest RBZ) to 82% (grass RBZ) due primarily to the annually recurring uniform amounts of the foregone income of cropland and other land rental.
- In all watersheds, all environmental indicators (except DO) due to the urban RBZ—widths showed large effects (>20% change). The mean normalized environmental indicators varied with the largest change in TN due to urban RBZs in Back Creek (60–95%), Sycamore Creek (37–91%), and Greens Mill (52–93%).
- All environmental indicators of wildlife RBZ widths showed relatively minor effects (≤5%) in all watersheds. The composite RBZ widths showed moderate effects (5–20%) in all environmental indicators except the DO in Back Creek.
- The holistic sustainability assessments revealed the least sustainable to most sustainable DMOs. The least sustainable and most sustainable DMOs (with the corresponding holistic sustainability scores) were, respectively, wildlife RBZ—11.4 m (0.54) and urban RBZ—45.8 m (1.00) in Back Creek, three-zone forest RBZ—8.6 m (0.32) and urban RBZ—45.8 m (1.00) in Sycamore Creek, and three-zone forest RBZ—8.6 m (0.62) and urban RBZ—45.8 m (1.00) in Greens Mill.
- Overall, the urban RBZ was found to be the most sustainable (1.00) across all watersheds.
- The holistic sustainability score tradeoffs have important policy implications for the U.S. government’s various riparian restoration and preservation programs including the USDA Conservation Reserve Program, which promotes the development of riparian buffers along streams [63]. Specifically, the results can help focus economic incentives and technical assistance based on the sustainability score tradeoffs of RBZ policy scenarios. The findings are also useful to inform landowners in the region who are considering implementing RBZs.
- The presented methodology is general enough to be applied to develop sustainable RBZ design strategy in the Southeast U.S. and beyond by obtaining appropriate data related to RBZ system components, vegetation types, and widths.

## Figures and Tables

**Table 1.** Description of baseline riparian buffer zone (RBZ) designs and assumptions for cost assessment (table modified from Ghimire et al.) [14].

Baseline RBZ (Average Width)	Description	National Resources Conservation Service (NRCS) Practice Scenarios and Assumptions for Cost Assessment [7]
Grass RBZ (8 m)	This buffer consisted of only grasses and forbs and was typically used along small streams and other drainages that flow through crop fields and pastures.	Practice 390—Riparian Herbaceous Cover Scenario #1: Warm Season Grass with Forbs. This scenario included establishment costs but specifically excluded opportunity costs. However, we added opportunity costs due to corn and soybeans cropping consistent with the NRCS’s other agricultural field Practice Scenarios. The opportunity cost due to land rental prices was estimated consistent with the United States Department of Agriculture (USDA) Conservation Reserve Program’s (CRP) annual rental payment rates [40].
Urban RBZ (23 m)	This buffer area consisted of Low-, Medium-, and High-Density Residential land use types.	Practice 393—Filter Strip Scenario #16—Filter Strip, Native species, Foregone Income. This Scenario included establishment costs and opportunity costs due to corn and soybean crops. In lieu of forgone income for the crops’ yields, land rental rates were applied in urban settings assuming that foregone income due to crops would be uncommon in urban settings. The opportunity cost due to land rental prices was estimated consistent with the USDA CRP annual rental payment rates.
Wildlife RBZ (46 m)	This buffer consisted of evergreen forest. Typical sites included former riparian forests, speculation property, or any non-forest condition which contained undesirable types of vegetation.	Practice: 391—Riparian Forest Buffer Scenario #11—Bare Root Hardwoods with tubes, 741 trees /hectare (300 trees /acre). This Scenario included establishment costs and opportunity costs (foregone income) due to corn crops. However, the opportunity costs were split between corn and soybeans consistent with all other case scenarios.
Naturalized RBZ (23 m)	This buffer consisted of forested wetlands. This was an inexpensive natural buffer that could be supplemented by interplanting tree and shrub seedlings as needed to achieve desired stocking densities.	Practice 391—Riparian Forest Buffer Scenario #14—Natural regeneration with some limited tree planting. This Scenario included the establishment costs of the buffer of trees and shrubs into a suitably prepared site located adjacent to and up-gradient from a watercourse or water body.
Three-zone forest RBZ (34 m)	This composite buffer consisted of three zones: zone 1 (4.6 m to 9.1 m wide undisturbed forest) that contained trees along the edge of the stream; zone 2 (9.1 m to 30.5 m wide managed forest) that filtered sediment and nutrients that passed through zone 3; zone 3 (6.1 m to 9.1 m wide grass strip).	This composite RBZ of three zones (zone 1, zone 2, and zone 3) included three Practice Scenarios: zone 1 (Practice 391—Riparian Forest Buffer Scenario #2—Bare-root, hand planted, conifers, hardwoods, shrubs); zone 2 (Practice 391—Riparian Forest Buffer Scenario #7—Shrub Planting, 1680 stems/hectare (680 stems/acre), no tubes); and zone 3 (Practice 390—Riparian Herbaceous Cover Scenario #1: Warm Season Grass with Forbs). All zone Scenarios included establishment costs and opportunity costs (foregone income) due to corn and soybean crops. The opportunity costs were split between corn and soybeans, consistent with all other scenarios. The average width of zone 1, zone 2, and zone 3 were 6.9 m, 19.8 m, and 7.6 m, respectively.
Two-zone forest RBZ (27 m)	A two-zone forest RBZ would be a modification to the three-zone forest RBZ with an elimination of zone 3.	This composite RBZ of two zones (zone 1 and zone 2) included two Practice Scenarios: zone 1 (Practice 391—Riparian Forest Buffer Scenario #2—Bare-root, hand planted, conifers, hardwoods, shrubs) and zone 2 (Practice 391—Riparian Forest Buffer Scenario #7—Shrub Planting, 1680 stems/hectare (680 stems/acre), no tubes). Both zone Scenarios included establishment costs and opportunity costs due to corn and soybean crops. The opportunity costs were split between corn and soybeans, consistent with all other scenarios.

**Table 2.** Description of the three sets of conceptual riparian buffer zone (RBZ) designs: 40, 32, and 48 decision management objectives (DMOs) in Back Creek, Sycamore Creek, and Greens Mill Run, respectively. The **Bold** are baseline DMOs. Naturalized RBZ was absent in Back Creek and grass RBZ and naturalized RBZ were absent in Sycamore Creek (adapted from Ghimire et al.) [14].

Back Creek RBZ DMOs—Width	DMO	Sycamore Creek RBZ DMOs—Width	DMO	Greens Mill Run RBZ DMOS—Width	DMO
Grass RBZ—1.9 m	DMO1	Urban RBZ—5.7 m	DMO1	Grass RBZ—1.9 m	DMO1
Grass RBZ—3.8 m	DMO2	Urban RBZ—11.5 m	DMO2	Grass RBZ—3.8 m	DMO2
Grass RBZ—5.7 m	DMO3	Urban RBZ—17.2 m	DMO3	Grass RBZ—5.7 m	DMO3
**Grass RBZ—7.6 m**	**DMO4**	**Urban RBZ—22.9 m**	**DMO4**	**Grass RBZ—7.6 m**	**DMO4**
Grass RBZ—9.5 m	DMO5	Urban RBZ—28.6 m	DMO5	Grass RBZ—9.5 m	DMO5
Grass RBZ—11.4 m	DMO6	Urban RBZ—34.4 m	DMO6	Grass RBZ—11.4 m	DMO6
Grass RBZ—13.3 m	DMO7	Urban RBZ—40.1 m	DMO7	Grass RBZ—13.3 m	DMO7
Grass RBZ—15.9 m	DMO8	Urban RBZ—45.8 m	DMO8	Grass RBZ—15.9 m	DMO8
Urban RBZ—5.7 m	DMO9	Three-zone forest RBZ—8.6 m	DMO9	Urban RBZ—5.7 m	DMO9
Urban RBZ—11.5 m	DMO10	Three-zone forest RBZ—17.2 m	DMO10	Urban RBZ—11.5 m	DMO10
Urban RBZ—17.2 m	DMO11	Three-zone forest RBZ—25.7 m	DMO11	Urban RBZ—17.2 m	DMO11
**Urban RBZ—22.9 m**	**DMO12**	**Three-zone forest RBZ**—**34.3 m**	**DMO12**	**Urban RBZ—22.9 m**	**DMO12**
Urban RBZ—28.6 m	DMO13	Three-zone forest RBZ—42.9 m	DMO13	Urban RBZ—28.6 m	DMO13
Urban RBZ—34.4 m	DMO14	Three-zone forest RBZ—51.5 m	DMO14	Urban RBZ—34.4 m	DMO14
Urban RBZ—40.1 m	DMO15	Three-zone forest RBZ—60 m	DMO15	Urban RBZ—40.1 m	DMO15
Urban RBZ—45.8 m	DMO16	Three-zone forest RBZ—68.6 m	DMO16	Urban RBZ—45.8 m	DMO16
Three-zone forest RBZ—8.6 m	DMO17	Two-zone forest RBZ—6.7 m	DMO17	Naturalized RBZ—5.7 m	DMO17
Three-zone forest RBZ—17.2 m	DMO18	Two-zone forest RBZ—13.4 m	DMO18	Naturalized RBZ—11.5 m	DMO18
Three-zone forest RBZ—25.7 m	DMO19	Two-zone forest RBZ—20 m	DMO19	Naturalized RBZ—17.2 m	DMO19
**Three-zone forest RBZ**—**34.3 m**	**DMO20**	**Two-zone forest RBZ**—**26.7 m**	**DMO20**	**Naturalized RBZ—22.9 m**	**DMO20**
Three-zone forest RBZ—42.9 m	DMO21	Two-zone forest RBZ—33.4 m	DMO21	Naturalized RBZ—28.6 m	DMO21
Three-zone forest RBZ—51.5 m	DMO22	Two-zone forest RBZ—40.1 m	DMO22	Naturalized RBZ—34.4 m	DMO22
Three-zone forest RBZ—60 m	DMO23	Two-zone forest RBZ—46.7 m	DMO23	Naturalized RBZ—40.1 m	DMO23
Three-zone forest RBZ—68.6 m	DMO24	Two-zone forest RBZ—53.4 m	DMO24	Naturalized RBZ—45.8 m	DMO24
Two-zone forest RBZ—6.7 m	DMO25	Wildlife RBZ—11.4 m	DMO25	Three-zone forest RBZ—8.6 m	DMO25
Two-zone forest RBZ—13.4 m	DMO26	Wildlife RBZ—22.9 m	DMO26	Three-zone forest RBZ—17.2 m	DMO26
Two-zone forest RBZ—20 m	DMO27	Wildlife RBZ—34.3 m	DMO27	Three-zone forest RBZ—25.7 m	DMO27
**Two-zone forest RBZ—26.7 m**	**DMO28**	**Wildlife RBZ**—**45.7 m**	**DMO28**	**Three-zone forest RBZ—34.3 m**	**DMO28**
Two-zone forest RBZ—33.4 m	DMO29	Wildlife RBZ—57.1 m	DMO29	Three-zone forest RBZ—42.9 m	DMO29
Two-zone forest RBZ—40.1 m	DMO30	Wildlife RBZ—68.6 m	DMO30	Three-zone forest RBZ—51.5 m	DMO30
Two-zone forest RBZ—46.7 m	DMO31	Wildlife RBZ—80 m	DMO31	Three-zone forest RBZ—60 m	DMO31
Two-zone forest RBZ—53.4 m	DMO32	Wildlife RBZ—91.4 m	DMO32	Three-zone forest RBZ—68.6 m	DMO32
Wildlife RBZ—11.4 m	DMO33			Two-zone forest RBZ—6.7 m	DMO33
Wildlife RBZ—22.9 m	DMO34			Two-zone forest RBZ—13.4 m	DMO34
Wildlife RBZ—34.3 m	DMO35			Two-zone forest RBZ—20 m	DMO35
**Wildlife RBZ—45.7 m**	**DMO36**			**Two-zone forest RBZ—26.7 m**	**DMO36**
Wildlife RBZ—57.1 m	DMO37			Two-zone forest RBZ—33.4 m	DMO37
Wildlife RBZ—68.6 m	DMO38			Two-zone forest RBZ—40.1 m	DMO38
Wildlife RBZ—80 m	DMO39			Two-zone forest RBZ—46.7 m	DMO39
Wildlife RBZ—91.4 m	DMO40			Two-zone forest RBZ—53.4 m	DMO40
				Wildlife RBZ—11.4 m	DMO41
				Wildlife RBZ—22.9 m	DMO42
				Wildlife RBZ—34.3 m	DMO43
				**Wildlife RBZ—45.7 m**	**DMO44**
				Wildlife RBZ—57.1 m	DMO45
				Wildlife RBZ—68.6 m	DMO46
				Wildlife RBZ—80 m	DMO47
				Wildlife RBZ—91.4 m	DMO48

**Table 3.** Summary of the unit net present value (NPV) costs for the six riparian buffer zones (RBZs). All values reported in $/hectare, estimated using the National Resources Conservation Service’s (NRCS) published values for the 2021 Fiscal Year [7]—also refer to Appendix A Table A6 for additional description on the items in the first column.

Cost Item	Three-Zone Forest RBZ		Two-Zone Forest RBZ		Grass RBZ		Urban RBZ		Wildlife RBZ		Naturalized RBZ	
	Cost (2021)	20-Y NPV	Cost (2021)	20-Y NPV	Cost (2021)	20-Y NPV	Cost (2021)	20-Y NPV	Cost (2021)	20-Y NPV	Cost (2021)	20-Y NPV
(E) Establishment cost subtotal	2184	2184	2590	2590	766	766	605	605	5812	5812	1453	1453
(O) Opportunity cost subtotal	620	8436	620	8436	620	8436	205	2787	620	8436	-	-
(M) Maintenance (10% of E)	217	2970	259	3521	77	1043	59	820	581	7897	146	1977
Total Unit NPV Costs ($/hectare) = E + O + M	3025	13,591	3472	14,547	1465	10,245	870	4213	7013	22,146	1599	3430
Annualized NPV cost ($/hectare)	-	1001	-	1070	-	754	-	309	-	1628	-	252

**Figure A4.**
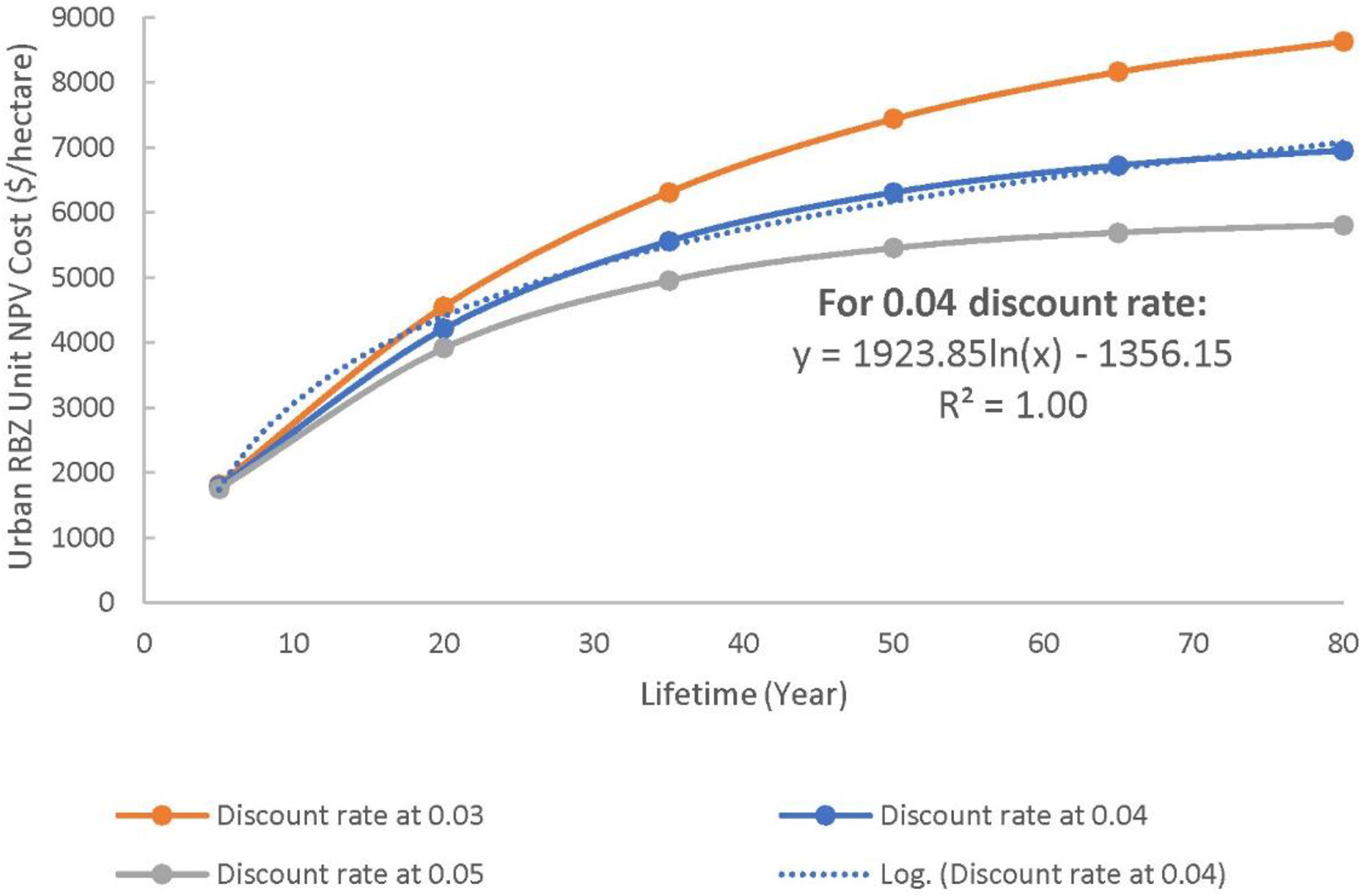
Sensitivity of the urban RBZ-lifetime to the unit NPV costs.

**Figure A5.**
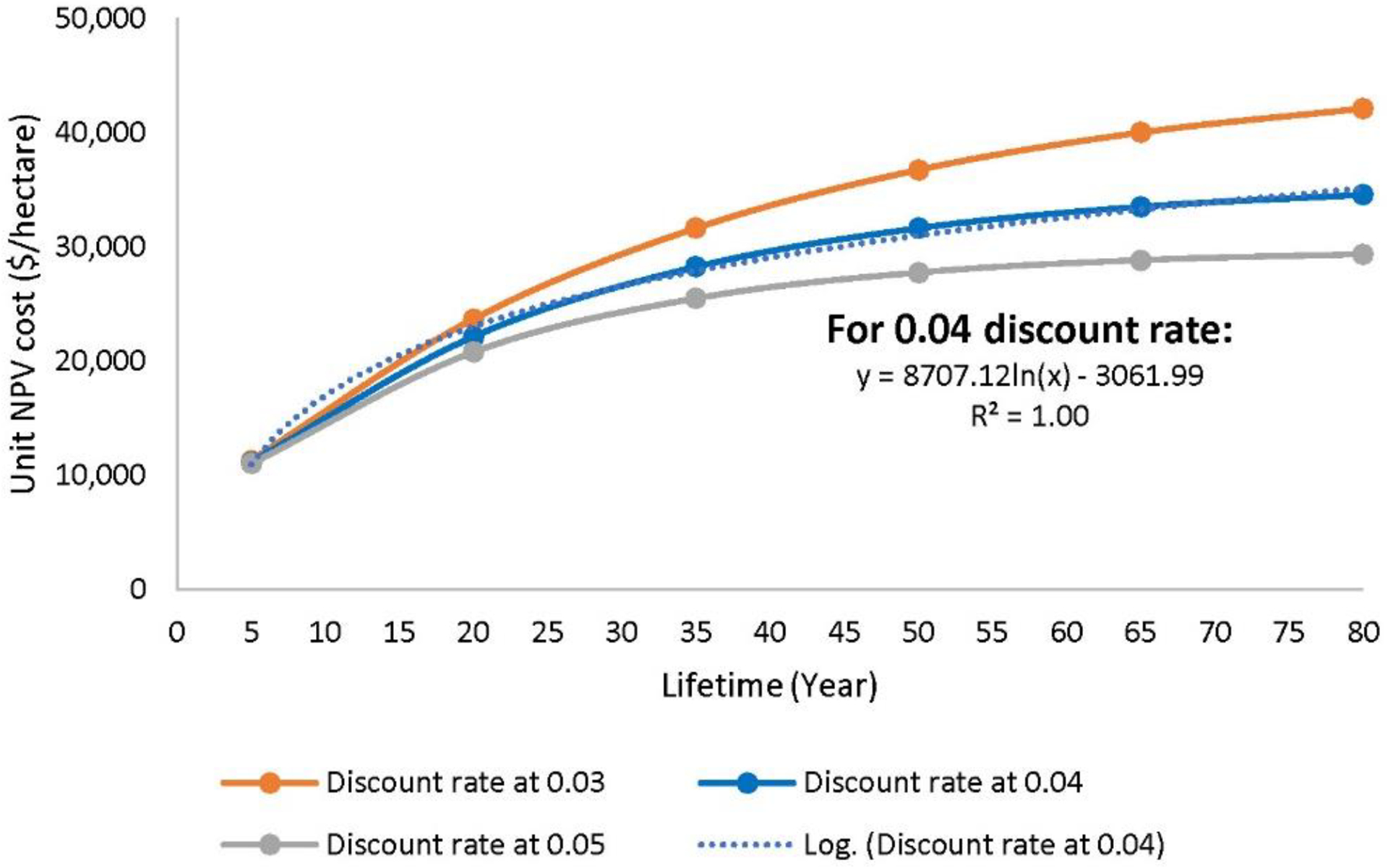
Sensitivity of wildlife RBZ-lifetime to the unit NPV costs.

## Data Availability

The data presented in this study are openly available within the manuscript and its Appendix A files. Additional data are published through the EPA ScienceHub (https://www.data.gov/).

## Appendix A

**Table A1.** Summary of previous studies that focused cost assessments of riparian buffer zones (RBZs).

Authors (Year)	Synthesis	Relevance to Current Work
Qiu and Prato (1998) [18]	They conducted an economic evaluation of riparian buffers in reducing instream non-point source pollutants from alternative farming systems in Missouri, USA. The alternative farming systems consisted of crop rotation, tillage system, fertilizer application rate, and pesticide application rate. They estimated RBZ costs (construction, maintenance, and opportunity) and government cost savings in the form of Conservation Reserve Program (CRP) rental payments. Their results supported government cost savings by maintaining the buffers.	They used the Soil and Water Assessment Tool (SWAT) to assess water quality indicators that included instream sediment yield, nitrogen concentration, and atrazine concentration. We developed the NPV cost calculation methods using opportunity, maintenance, and establishment costs data published by the Natural Resources Conservation Service (NRCS). We used watershed-wide RBZ WQI parameters that were estimated by Ghimire, et al. [14] using the Hydrologic and Water Quality System (HAWQS) that employed SWAT as modeling engine.
Rickerl et al. (2000) [59]	They evaluated the economic, agronomic, and environmental performance of buffered and unbuffered wetlands, five crop management scenarios, and three crop farming systems in South Dakota, USA. They suggested both economic and environmental benefits of enrolling in Wetland Reserve Programs (WRPs).	They collected wetland surface water quality sampling (nitrogen and phosphorous samples) and estimated net returns to buffer strips using published rates of government subsidies. We used five WQI parameters (DO, TP, TN, TS, and BD) as environmental indicators estimated by Ghimire et al. [14].
Bonham et al. (2006) [27]	They assessed compliance costs to farms by comparing three policy scenarios (baseline, nutrient management, and buffer scenarios) in a livestock-intensive watershed in Virginia, USA. The compliance cost was reported as the revenue difference between the baseline scenario and the alternative practice scenario.	We estimated NPV costs that included the establishment, maintenance, and opportunity costs using the cost data from the NRCS.
Frimpong et al. (2006) [45]	They assessed the cost effectiveness of vegetative filter strips and instream half-log structures for increasing the Index of Biotic Integrity as a measure of different stream-order health in a watershed in Indiana, USA. They used a probabilistic approach to assign riparian land values of different stream orders.	They determined the land rental values from land transaction data and determined establishment and maintenance costs based on the NRCS. They estimated maintenance costs to be approximately 10% of establishment costs and social discount rates (1, 3, and 5 percent). We approximated maintenance costs to be 10% of the establishment costs and used the average discount rate of 4%.
LeDoux (2006) [54]	They performed opportunity cost assessment of streamside forest buffer zones considering four logging technologies, two hardwood stands, and three types of riparian buffer zones. They demonstrated forgoing timber harvesting revenue to be sizeable opportunity costs.	They conducted economic analysis using a real interest rate of 4% that was adapted for our NPV costs analysis.
Roberts et al. (2009) [19]	They evaluated annualized costs that included the opportunity costs, the capital costs, and maintenance costs of 45.7 m wide agricultural buffer strips in a watershed situated in Tennessee, USA.	They calculated an annualized aggregate cost at $262/ha ($106/acre).
Qiu and Dosskey (2012) [20]	They evaluated cost effectiveness of conservation buffer placement strategies in landscapes, including those based on soil surveys, topography, and riparian-focused, in a watershed in New Jersey, USA. They found that the riparian strategies were cost-effective compared to the rest and suggested alternative placement strategies, especially when riparian-focus strategies could not meet environmental goals.	They calculated cost-effectiveness as the ratio of aggregated potential environmental benefits (reducing soil erosion, enhancing wildlife habitat, and mitigating stormwater impacts) to the costs of establishing and maintaining the conservation buffers in agricultural land.
Carvajal and Janmaat (2016) [17]	They proposed to perform cost-benefit analysis of riparian rehabilitation projects along a small creek in British Columbia, Canada using recorded-survey data of positive impacts on land productivity and ecosystem services including riparian habitat restoration, improvement in outdoor recreation, improvements in soil retention and erosion control, water quality, fish habitat enhancement, and climate regulation.	They used Benefit Transfer methodology, i.e., using the results with adjustment of similar analyses conducted elsewhere, to create values for the ecosystem services. They obtained the Willingness to Pay (WTP) values from similar studies and transformed by area and discounted with the time. The lifetime of the project was taken as 20 years with the 3% and 5% discount rates.

**Table A2.** Actual environmental indicator values (mg/L) and economic indicator (NPV costs) values ($) of each decision management objective (DMO) in Back Creek. Refer to Table 2 for DMO description.

DMO	DO (mg/L)	TP (mg/L)	TN (mg/L)	TS (mg/L)	BD (mg/L)	NPV Costs ($)
DMO1	9.6	0.2	2.0	12.5	0.5	77,837
DMO2	9.6	0.2	2.0	12.4	0.5	155,674
DMO3	9.6	0.2	2.0	12.4	0.5	233,512
DMO4	9.6	0.2	2.0	12.3	0.5	311,349
DMO5	9.6	0.2	2.0	12.3	0.5	389,186
DMO6	9.6	0.2	2.0	12.3	0.5	467,023
DMO7	9.6	0.2	2.0	12.2	0.5	544,860
DMO8	9.6	0.2	2.0	12.2	0.5	622,697
DMO9	9.8	0.1	1.7	6.1	0.3	96,043
DMO10	9.9	0.1	1.6	4.7	0.3	193,770
DMO11	9.9	0.1	1.5	4.3	0.2	289,813
DMO12	10.0	0.1	1.4	3.9	0.2	385,855
DMO13	10.0	0.1	1.3	3.6	0.2	481,898
DMO14	10.0	0.1	1.2	3.6	0.2	579,625
DMO15	10.0	0.1	1.2	3.6	0.2	675,668
DMO16	10.0	0.1	1.1	3.6	0.2	771,710
DMO17	9.7	0.2	1.9	11.1	0.3	467,408
DMO18	9.7	0.2	1.9	11.1	0.3	934,816
DMO19	9.7	0.2	1.9	11.1	0.3	1,396,790
DMO20	9.7	0.2	1.9	11.2	0.3	1,864,198
DMO21	9.7	0.2	1.9	11.3	0.3	2,331,606
DMO22	9.7	0.2	1.9	11.4	0.3	2,799,014
DMO23	9.7	0.2	1.8	11.5	0.3	3,260,987
DMO24	9.7	0.2	1.8	11.5	0.4	3,728,396
DMO25	9.7	0.2	2.0	11.0	0.4	389,762
DMO26	9.7	0.2	2.0	10.6	0.3	779,524
DMO27	9.7	0.2	2.0	10.3	0.3	1,163,469
DMO28	9.7	0.2	2.0	10.1	0.3	1,553,231
DMO29	9.7	0.2	1.9	10.0	0.3	1,942,994
DMO30	9.7	0.2	1.9	10.0	0.3	2,332,756
DMO31	9.7	0.2	1.9	10.0	0.3	2,716,701
DMO32	9.7	0.2	1.9	10.0	0.3	3,106,463
DMO33	9.6	0.2	2.0	12.7	0.5	1,009,576
DMO34	9.6	0.2	2.0	12.7	0.5	2,028,008
DMO35	9.6	0.2	2.0	12.7	0.5	3,037,585
DMO36	9.6	0.2	2.0	12.7	0.5	4,047,161
DMO37	9.6	0.2	2.0	12.7	0.5	5,056,737
DMO38	9.6	0.2	2.0	12.7	0.5	6,075,169
DMO39	9.6	0.2	2.0	12.7	0.5	7,084,745
DMO40	9.6	0.2	2.0	12.7	0.5	8,094,322
Average	9.7	0.2	1.8	10.1	0.4	1,836,948

**Table A3.** Actual environmental indicator values (mg/L) and economic indicator (NPV costs) values ($) of each decision management objective (DMO) in Sycamore Creek. Refer to Table 2 for DMO description.

DMO	DO (mg/L)	TP (mg/L)	TN (mg/L)	TS (mg/L)	BD (mg/L)	NPV Costs ($)
DMO1	3.9	0.1	1.0	7.5	0.2	28,988
DMO2	4.0	0.1	0.9	5.0	0.1	58,485
DMO3	4.0	0.1	0.8	3.2	0.1	87,473
DMO4	4.1	0.1	0.7	1.9	0.0	116,461
DMO5	4.1	0.1	0.6	0.7	0.0	145,449
DMO6	4.1	0.1	0.5	0.6	0.0	174,946
DMO7	4.1	0.1	0.5	0.6	0.0	203,934
DMO8	4.1	0.1	0.4	0.6	0.0	232,922
DMO9	3.7	0.2	1.3	19.4	0.5	141,076
DMO10	3.7	0.2	1.3	19.4	0.5	282,151
DMO11	3.7	0.2	1.3	19.4	0.5	421,587
DMO12	3.7	0.2	1.3	19.4	0.5	562,662
DMO13	3.7	0.2	1.3	19.4	0.5	703,738
DMO14	3.7	0.2	1.2	19.4	0.5	844,814
DMO15	3.7	0.2	1.2	19.4	0.5	984,249
DMO16	3.7	0.2	1.2	19.5	0.5	1,125,325
DMO17	3.7	0.2	1.3	19.3	0.5	117,640
DMO18	3.7	0.2	1.3	19.3	0.5	235,280
DMO19	3.7	0.2	1.3	19.3	0.5	351,165
DMO20	3.7	0.2	1.3	19.3	0.5	468,805
DMO21	3.7	0.2	1.3	19.3	0.5	586,445
DMO22	3.7	0.2	1.2	19.3	0.5	704,085
DMO23	3.7	0.2	1.2	19.3	0.5	819,969
DMO24	3.7	0.2	1.2	19.3	0.5	937,610
DMO25	3.7	0.2	1.3	19.3	0.5	304,716
DMO26	3.7	0.2	1.3	19.3	0.5	612,105
DMO27	3.7	0.2	1.3	19.3	0.5	916,820
DMO28	3.7	0.2	1.3	19.3	0.5	1,221,536
DMO29	3.7	0.2	1.3	19.3	0.5	1,526,252
DMO30	3.7	0.2	1.3	19.3	0.5	1,833,641
DMO31	3.7	0.2	1.3	19.3	0.5	2,138,357
DMO32	3.7	0.2	1.3	19.3	0.5	2,443,072
Average	3.8	0.2	1.1	15.1	0.4	666,617

**Table A4.** Actual environmental indicator values (mg/L) and economic indicator (NPV costs) values ($) of each decision management objective (DMO) in Greens Mill Run. Refer to Table 2 for DMO description.

DMO	DO (mg/L)	TP (mg/L)	TN (mg/L)	TS (mg/L)	BD (mg/L)	NPV Costs ($)
DMO1	6.8	0.4	3.3	60.9	2.2	44,651
DMO2	6.8	0.4	3.3	60.9	2.2	89,302
DMO3	6.8	0.4	3.3	60.9	2.2	133,952
DMO4	6.8	0.4	3.3	60.9	2.2	178,603
DMO5	6.8	0.4	3.3	60.9	2.2	223,254
DMO6	6.8	0.4	3.3	60.9	2.2	267,905
DMO7	6.8	0.4	3.3	60.9	2.2	312,556
DMO8	6.8	0.4	3.3	60.9	2.2	357,206
DMO9	7.1	0.4	2.9	39.9	1.5	55,094
DMO10	7.2	0.4	2.6	33.9	1.3	111,155
DMO11	7.3	0.4	2.4	29.4	1.2	166,249
DMO12	7.4	0.4	2.2	26.2	1.1	221,343
DMO13	7.4	0.4	2.1	23.6	1.0	276,437
DMO14	7.4	0.4	1.9	23.3	1.0	332,498
DMO15	7.4	0.4	1.7	23.3	1.0	387,592
DMO16	7.4	0.4	1.6	23.3	1.0	442,687
DMO17	6.8	0.4	3.3	60.9	2.2	44,854
DMO18	6.8	0.4	3.3	60.9	2.2	90,495
DMO19	6.8	0.4	3.3	60.9	2.2	135,349
DMO20	6.8	0.4	3.3	60.9	2.2	180,203
DMO21	6.8	0.4	3.3	60.9	2.2	225,057
DMO22	6.8	0.4	3.3	60.9	2.2	270,698
DMO23	6.8	0.4	3.2	60.9	2.2	315,552
DMO24	6.8	0.4	3.2	60.9	2.2	360,406
DMO25	6.8	0.4	3.3	61.0	2.2	268,126
DMO26	6.8	0.4	3.3	61.0	2.2	536,251
DMO27	6.8	0.4	3.3	61.0	2.2	801,259
DMO28	6.8	0.4	3.3	61.0	2.2	1,069,385
DMO29	6.8	0.4	3.3	61.1	2.2	1,337,510
DMO30	6.8	0.4	3.3	61.1	2.2	1,605,636
DMO31	6.8	0.4	3.3	61.1	2.2	1,870,644
DMO32	6.8	0.4	3.3	61.1	2.2	2,138,770
DMO33	6.8	0.4	3.3	61.0	2.2	223,585
DMO34	6.8	0.4	3.3	61.0	2.2	447,169
DMO35	6.8	0.4	3.3	61.0	2.2	667,416
DMO36	6.8	0.4	3.3	61.0	2.2	891,001
DMO37	6.8	0.4	3.3	61.0	2.2	1,114,586
DMO38	6.8	0.4	3.3	61.0	2.2	1,338,170
DMO39	6.8	0.4	3.3	61.0	2.2	1,558,417
DMO40	6.8	0.4	3.3	61.0	2.2	1,782,002
DMO41	6.8	0.4	3.3	61.0	2.2	579,137
DMO42	6.8	0.4	3.3	61.0	2.2	1,163,354
DMO43	6.8	0.4	3.3	61.0	2.2	1,742,490
DMO44	6.8	0.4	3.3	61.0	2.2	2,321,627
DMO45	6.8	0.4	3.3	61.0	2.2	2,900,764
DMO46	6.8	0.4	3.3	61.0	2.2	3,484,981
DMO47	6.8	0.4	3.3	61.0	2.2	4,064,118
DMO48	6.8	0.4	3.3	61.0	2.2	4,643,254
Average	6.9	0.4	3.1	55.4	2.0	911,932

**Table A5.** Description of the unit net present value (NPV) costs for the six baseline riparian buffer zones (RBZs). All values reported in $/hectare, estimated using the National Resources Conservation Service’s (NRCS) published values for the 2021 Fiscal Year [7]. Refer also to Table A6 for additional description on the items in the first column.

Item	Three-Zone Forest RBZ		Two-Zone Forest RBZ		Grass RBZ		Urban RBZ		Wildlife RBZ		Naturalized RBZ	
	Cost (2021)	20-Y NPV	Cost (2021)	20-Y NPV	Cost (2021)	20-Y NPV	Cost (2021)	20-Y NPV	Cost (2021)	20-Y NPV	Cost (2021)	20-Y NPV
Tractor, agricultural, 60 HP	-	-	-	-	-	-	-	-	-	-	59	59
Chemical, ground application	7	7	-	-	30	30	-	-	-	-	282	282
Tillage, Light	-	-	-	-	-	-	77	77	-	-	-	-
Seeding Operation, No Till/Grass Drill	12	12	-	-	54	54	54	54	-	-	-	-
All-terrain vehicles	-	-	-	-	-	-	82	82	-	-	-	-
Truck, Pickup	7	7	10	10	-	-	-	-	-	-	-	-
Trailer, enclosed, small	2	2	5	5	-	-	-	-	-	-	-	-
Hand tools, tree planting	86	86	114	114	-	-	-	-	136	136	-	-
Tree shelter, solid tube type, 127 mm. 127 mm × 1219 mm	-	-	-	-	-	-	-	-	2750	2750	230	230
Cable ties, plastic	-	-	-	-	-	-	-	-	89	89	-	-
Stakes, wood, 19 mm × 19 mm × 1524 mm	-	-	-	-	-	-	-	-	1112	1112	86	86
Herbicide, Glyphosate	10	10	-	-	44	44	-	-	-	-	-	-
Native Perennial Grasses, Medium Density	141	141	-	-	640	640	393	393	-	-	195	195
Shrub, Seedling, Medium	1651	1651	2120	2120	-	-	-	-	-	-	-	-
Tree, Conifer, Seedling, Small	20	20	27	27	-	-	-	-	-	-	-	-
Tree, Hardwood, Seedling, Medium	49	49	62	62	-	-	-	-	1127	1127	173	173
General Labor	198	198	252	252	-	-	-	-	516	516	430	430
Supervisor/Manager	-	-	-	-	-	-	-	-	82	82	-	-
(E) Establishment cost subtotal	2184	2184	2590	2590	766	766	605	605	5812	5812	1453	1453
Corn Dryland annual foregone income per hectare	230	3126	230	3126	230	3126	-	-	230	3126	-	-
Soybeans Dryland annual foregone income per hectare	185	2520	185	2520	185	2520	-	-	185	2520	-	-
North Carolina land rental	205	2787	205	2787	205	2787	205	2787	205	2787	-	-
(O) Opportunity cost subtotal	620	8436	620	8436	620	8436	205	2787	620	8436	-	-
(M) Maintenance (10% of *E*)	217	2970	259	3521	77	1043	59	820	581	7897	146	1977
Total Unit NPV Costs ($/hectare) = E + O + M	3025	13,591	3472	14,547	1465	10,245	870	4213	7013	22,146	1599	3430
Annualized NPV cost ($/hectare)	-	1001	-	1070	-	754	-	309	-	1628	-	252

**Table A6.** Description of the items used in cost calculation of the riparian buffer zones [7].

Establishment Cost Item	Description
Tractor, agricultural, 60 HP	Equipment and power unit costs, except labor.
Chemical, ground application	Includes equipment, power unit, and labor costs.
Tillage, Light	Includes light disking (tandem) or field cultivator, equipment, power unit and labor costs.
Seeding Operation, No Till/Grass Drill	Includes equipment, power unit, and labor costs.
All-terrain vehicles	Includes equipment, power unit, and labor costs.
Truck, Pickup	Equipment and power unit costs, 2/3 h (labor not included) @$25/hour.
Trailer, enclosed, small	Typically, less than 76.2 cm length pulled by a pickup to transport materials and equipment, 0.3 hectare (truck not included) @ $27/hectare.
Hand tools, tree planting	Various hand tools for digging holes and planting trees such as augers, dibble bars, planting shovel, hoe-dad (equipment only, labor not included) @$11.6/hour.
Tree shelter, solid tube type, 127 mm. 127 mm × 1219 mm	Solid tube type for protection from animal damage; materials only.
Cable ties, plastic (typical 203–305 mm)	Plastic cable ties to assist in securing items (typical 203–305 mm); materials only.
Stakes, wood, 19 mm × 19 mm × 1524 mm	Wood stakes to fasten items in place; materials only.
Herbicide, Glyphosate	A broad-spectrum, non-selective systemic herbicide; materials and shipping only.
Native Perennial Grasses, Medium Density	A mix of native perennial grasses, legumes, and/or forbs, grasses typically greater than 50% of the mix; planted at medium to higher density (441–646 pure live seeds/m^2^); material and shipping only.
Shrub, Seedling, Medium	Bare root shrub seedling, 457 to 914 mm (18 to 36 in) tall; includes tropical containerized seedlings 164 to 328 cm^3^; includes materials and shipping only; @$1.4/unit.
Tree, Conifer, Seedling, Small	Containerized, 66 to 98 cm^3^; or bare root conifer seedlings (one-year old seedlings grown in their original seedbed), includes materials and shipping only; 199 units/hectare @ $0.5/unit.
Tree, Hardwood, Seedling, Medium	Bare root shrub seedling, 457 to 1524 mm tall; includes tropical containerized seedlings 164 to 328 cm^3^ (10 to 20 in^3^); includes materials and shipping only; @ $1.5/unit.
General Labor	Labor using basic tools such as power tool, shovels, and other tools that do not require extensive training; examples are herder, concrete placement, materials spreader, flagger; @ $21.7/hour.
Supervisor/Manager	Includes crew supervisors, foremen and farm/ranch managers time required for adopting new technology.
Opportunity cost item	Description
Corn Dryland annual foregone income	Dryland Corn is primary crop; 0.2 hectare @ $460/hectare.
Soybeans Dryland annual foregone income	Dryland Soybeans is primary crop; 0.2 hectare @ $371/hectare.
North Carolina land rental costs	Average land rental cost; 0.4 hectare @ $205/hectare.

### Example of DEA Formulation

The DEA formulation of DMO1 in Back Creek is provided as an example using corresponding mean-normalized values (Figure 2); all other DMOs’ DEAs follow the similar form with corresponding *w*_*i*_ and mean-normalized value:

For DMO1, minimize:

(A1)
$$E_{\text{DMO}1}^{-1}=\frac{1}{0.04}\left(1.01w_{1}+1.07w_{2}+1.09w_{3}+1.23w_{4}+1.30w_{5}\right)$$

subject to:

(A2)
$$\frac{1}{0.04}\left(1.01w_{1}+1.07w_{2}+1.09w_{3}+1.23w_{4}+1.30w_{5}\right)\geq 1$$

(A3)
$$\frac{1}{0.04}\left(1.01w_{1}+1.07w_{2}+1.09w_{3}+1.23w_{4}+1.29w_{5}\right)\geq 1$$

(A4)
$$\frac{1}{0.04}\left(1.01w_{1}+1.07w_{2}+1.08w_{3}+1.22w_{4}+1.29w_{5}\right)\geq 1$$

(A5)
$$\frac{1}{0.04}\left(1.01w_{1}+1.08w_{2}+1.09w_{3}+1.25w_{4}+1.30w_{5}\right)\geq 1$$

(A6)
$$w_{1},w_{2},\ldots w_{X}\geq 0$$

Additional weighting schemes, the equal weights, and unequal weights were employed as shown in Equations (A7) and (A8), respectively:

(A7)
$$W_{1}=w_{2}=\ldots ..=w_{X};$$

(A8)
$$w_{4}\geq w_{3}\geq w_{5}\geq w_{2}\geq w_{1}.$$

Here, the weights corresponded as: *w*_1_ = DO^−1^; *w*_2_ = TP; *w*_3_ = TN; *w*_4_ = TS; and *w*_5_ = BD. Note that the mean normalized DO values were inverted (DO^−1^) to be consistent with other environmental indicators.

## References

[1] USEPA. Impaired Waters and TMDLs. Available online: https://www.epa.gov/tmdl/overview-identifying-and-restoring-impaired-waters-under-section-303d-cwa (accessed on 29 September 2020).

[2] Cole LJ; Stockan J; Helliwell R Managing riparian buffer strips to optimise ecosystem services: A review. Agric. Ecosyst. Environ 2020, 296, 106891.

[3] Fischer RA; Martin CO; Fischenich J Improving riparian buffer strips and corridors for water quality and wildlife. In Proceedings of the International Conference on Riparian Ecology and Management in Multi-Land Use Watersheds, Portland, OR, USA, 28–31 August 2000.

[4] Mander Ü; Hayakawa Y; Kuusemets V Purification processes, ecological functions, planning and design of riparian buffer zones in agricultural watersheds. Ecol. Eng 2005, 24, 421–432.

[5] USEPA. Riparian Buffer Width, Vegetative Cover, and Nitrogen Removal Effectiveness: A Review of Current Science And Regulations; U.S. Environmental Protection Agency: Cincinnati, OH, USA, 2005.

[6] Fischer RA; Fischenich JC Design Recommendations for Riparian Corridors and Vegetated Buffer Strips; U.S. Army Corps of Engineers: Vicksburg, MS, USA, 2000.

[7] USDA-NRCS. North Carolina Payment Schedules. Available online: https://www.nrcs.usda.gov/wps/portal/nrcs/detail/national/programs/financial/?cid=nrcseprd1328255 (accessed on 22 October 2021).

[8] Mayer PM; Reynolds SK Jr.; McCutchen MD; Canfield TJ Meta-analysis of nitrogen removal in riparian buffers. J. Environ. Qual 2007, 36, 1172–1180. 17596626 10.2134/jeq2006.0462

[9] Valkama E; Usva K; Saarinen M; Uusi-Kämppä J A Meta-Analysis on Nitrogen Retention by Buffer Zones. J. Environ. Qual 2019, 48, 270–279. 30951137 10.2134/jeq2018.03.0120

[10] Blankenberg A-GB; Skarbøvik E Phosphorus retention, erosion protection and farmers’ perceptions of riparian buffer zones with grass and natural vegetation: Case studies from South-Eastern Norway. Ambio 2020, 49, 1838–1849. 32930956 10.1007/s13280-020-01361-5 PMC7502646

[11] Vigiak O; Malagó A; Bouraoui F; Grizzetti B; Weissteiner CJ; Pastori M Impact of current riparian land on sediment retention in the Danube River Basin. Sustain. Water Qual. Ecol 2016, 8, 30–49.

[12] Uusi-Kämppä J; Braskerud B; Jansson H; Syversen N; Uusitalo R Buffer zones and constructed wetlands as filters for agricultural phosphorus. J. Environ. Qual 2000, 29, 151–158.

[13] Cooper J; Gilliam J; Daniels R; Robarge W Riparian areas as filters for agricultural sediment. Soil Sci. Soc. Am. J 1987, 51, 416–420.

[14] Ghimire SR; Corona J; Parmar R; Mahadwar G; Srinivasan R; Mendoza K; Johnston JM Sensitivity of Riparian Buffer Designs to Climate Change—Nutrient and Sediment Loading to Streams: A Case Study in the Albemarle-Pamlico River Basins (USA) using HAWQS. Sustainability 2021, 13, 12380. 10.3390/su132212380 PMC8765004 35059223

[15] Trenholm R; Lantz V; Martínez-Espiñeira R; Little S Cost-benefit analysis of riparian protection in an eastern Canadian watershed. J. Environ. Manag 2013, 116, 81–94. 10.1016/j.jenvman.2012.11.039 23291404

[16] Chang C-L; Ying-Sung H; Bing-Jean L; Chuan-Yi W; Li-Jung W A cost-benefit analysis for the implementation of riparian buffer strips in the Shihmen reservoir watershed. Int. J. Sed. Res 2011, 26, 395–401.

[17] Carvajal V; Janmaat J A Cost-Benefit Analysis of a Riparian Rehabilitation Project on Alderson Creek, Township of Spallumcheen; University of British Columbia: Vancouver, BC, Canada; Kelowna, BC, Canada, 2016.

[18] Qiu Z; Prato T Economic evaluation of riparian buffers in an agricultural watershed. J. Am. Water Resour. Assoc 1998, 34, 877–890.

[19] Roberts DC; Clark CD; English BC; Park WM; Roberts RK Estimating annualized riparian buffer costs for the Harpeth River watershed. Appl. Econo. Perspec. Pol 2009, 31, 894–913.

[20] Qiu Z; Dosskey M Multiple function benefit–Cost comparison of conservation buffer placement strategies. Landsc. Urban Plan 2012, 107, 89–99.

[21] Tiwari T; Lundström J; Kuglerová L; Laudon H; Öhman K; Ågren A Cost of riparian buffer zones: A comparison of hydrologically adapted site-specific riparian buffers with traditional fixed widths. Water Resour. Res 2016, 52, 1056–1069.

[22] Hecht AD; Fiksel J; Moses M Working toward a sustainable future. Sustain. Sci. Pract. Policy 2014, 10, 65–75.

[23] Liu J; Mooney H; Hull V; Davis SJ; Gaskell J; Hertel T; Lubchenco J; Seto KC; Gleick P; Kremen C Systems integration for global sustainability. Science 2015, 347, 1258832. 25722418 10.1126/science.1258832

[24] Ghimire SR; Johnston JM A modified eco-efficiency framework and methodology for advancing the state of practice of sustainability analysis as applied to green infrastructure. Integr. Environ. Assess. Manag 2017, 13, 821–831. 28304134 10.1002/ieam.1928 PMC6093199

[25] Allen T; Tainter J; Hoekstra T Supply-Side Sustainability; Columbia University Press: New York, NY, USA, 2003.

[26] Ghimire SR; Johnston JM Sustainability assessment of agricultural rainwater harvesting: Evaluation of alternative crop types and irrigation practices. PLoS ONE 2019, 14, e0216452. 31075147 10.1371/journal.pone.0216452 PMC6510416

[27] Bonham JG; Bosch DJ; Pease JW Cost-effectiveness of nutrient management and buffers: Comparisons of two spatial scenarios. J. Agric. Appl. Econ 2006, 38, 17–32.

[28] ISO 14044; Environmental Management—Life Cycle Assessment—Requirements and Guidelines. International Organization for Standardization: Geneva, Switzerland, 2006.

[29] ISO 14040; Environmental Management—Life Cycle Assessment—Principles and Framework. International Organization for Standardization: Geneva, Switzerland, 2006.

[30] Liang X; van Dijk MP Economic and financial analysis on rainwater harvesting for agricultural irrigation in the rural areas of Beijing. Res. Conserv. Recy 2011, 55, 1100–1108.

[31] ISO 14040; Environmental Management—Life Cycle Assessment—General Principles and Framework, 2 ed. International Organization for Standardization: Geneva, Switzerland, 1997; p. 20.

[32] Xiao GJ; Zhang QA; Xiong YC; Lin MZ; Wang J Integrating rainwater harvesting with supplemental irrigation into rain-fed spring wheat farming. Soil Tillage Res. 2007, 93, 429–437.

[33] Fuller S; Petersen S Life-cycle costing manual for the federal energy management program. In NIST Handbook; NIST Pubs.: Gaithersburg, MD, USA, 1996; p. 135.

[34] Farrell MJ The measurement of productive efficiency. J. Roy. Stat. Soc. Ser. A 1957, 120, 253–281.

[35] Charnes A; Cooper WW; Rhodes E Measuring the efficiency of decision making units. Europ. J. Opera. Res 1978, 2, 429–444.

[36] Zhang B; Bi J; Fan Z; Yuan Z; Ge J Eco-efficiency analysis of industrial system in China: A data envelopment analysis approach. Ecol. Econ 2008, 68, 306–316.

[37] Picazo-Tadeo AJ; Gómez-Limón JA; Reig-Martínez E Assessing farming eco-efficiency: A Data Envelopment Analysis approach. J. Environ. Manag 2011, 92, 1154–1164. 10.1016/j.jenvman.2010.11.025 21193265

[38] Kuosmanen T; Kortelainen M Measuring Eco-efficiency of Production with Data Envelopment Analysis. J. Ind. Ecol 2005, 9, 59–72.

[39] Schmidheiny S; Stigson B Eco-Efficiency: Creating More Value with Less Impact; World Business Council for Sustainable Development: Geneva, Switzerland, 2000.

[40] USDA-FSA. Conservation Reserve Program Statistics. Available online: https://www.fsa.usda.gov/Assets/USDA-FSA-Public/usdafiles/Conservation/PDF/Summary%20JUNE%202021%20CRPMonthly.pdf (accessed on 27 October 2021).

[41] USEPA. HAWQS (Version 2.0 DEV). Available online: https://dev.hawqs.tamu.edu/#/ (accessed on 26 July 2022).

[42] TAMU. Soil & Water Assessment Tool. Available online: https://swat.tamu.edu/ (accessed on 30 September 2020).

[43] Arnold J; Kiniry J; Srinivasan R; Williams J; Haney E; Neitsch S Soil & water assessment tool: Input/output documentation. Tex. Water Resour. Inst. Tech. Rep 2012, TR-439, 650.

[44] USDA. State Soil Geographic Data. Available online: https://sdmdataaccess.nrcs.usda.gov/ (accessed on 3 May 2021).

[45] Frimpong EA; Lee JG; Sutton TM Cost effectiveness of vegetative filter strips and instream half-logs for ecological restoration. J. Am. Water Resour. Assoc 2006, 42, 1349–1361.

[46] Commission, J.R.C.-E. Handbook on Constructing Composite Indicators: Methodology and User Guide; OECD Publishing: Washington, DC, USA, 2008.

[47] Juwana I; Muttil N; Perera B Indicator-based water sustainability assessment—A review. Sci. Total Environ 2012, 438, 357–371. 23022721 10.1016/j.scitotenv.2012.08.093

[48] Sarkis J Preparing your data for DEA. In Modeling Data Irregularities and Structural Complexities in Data Envelopment Analysis; Zhu J, Cook WD, Eds.; Springer: New York, NY, USA, 2007; pp. 305–320.

[49] Countryman D; Murrow J Economic analysis of contour tree buffer strips using present net value. J. Soil Water Conserv 2000, 55, 152–160.

[50] Gloria TP; Lippiatt BC; Cooper J Life cycle impact assessment weights to support environmentally preferable purchasing in the united states. Environ. Sci. Technol 2007, 41, 7551–7557. 18044540 10.1021/es070750+

[51] Goedkoop M; Spriensma R The Eco-Indicator 99: A Damage-Oriented Method for Life Cycle Impact Assessment, Methodology Report; PRé Consultants: Amersfoort, The Netherlands, 2000.

[52] Sironen S; Seppälä J; Leskinen P Towards more non-compensatory sustainable society index. Environ. Dev. Sustain 2014, 17, 587–621.

[53] Tyndall J; Bowman T Iowa Nutrient Reduction Strategy Best Management Practice Cost Overview Series: Constructed Wetlands; Department of Ecology & Natural Resource Management, Lowa State University: Ames, IA, USA, 2016.

[54] LeDoux CB Assessing the opportunity cost of implementing streamside management zone guidelines in eastern hardwood forests. For. Prod. J 2006, 56, 40–44.

[55] Bank, T.W. Urban Development Overview. Available online: https://www.worldbank.org/en/topic/urbandevelopment/overview#1 (accessed on 12 May 2022).

[56] House, T.W. White House Action Plan on Global Water Security; The White House: Washington, DC, USA, 2022.

[57] Shaffer R; Haney H; Worrell E; Aust W Forestry BMP implementation costs for Virginia. For. Prod. J 1998, 48, 9.

[58] UN. Welcome to the Sustainable Development Goal indicators website. Available online: https://unstats.un.org/sdgs (accessed on 6 September 2022).

[59] Rickerl D; Janssen L; Woodland R Buffered wetlands in agricultural landscapes in the Prairie Pothole region: Environmental, agronomic, and economic evaluations. J. Soil Water Conserv 2000, 55, 220–225.

[60] He Y; Wang P; Sheng H; Wang D; Huang M; Cao C Sustainability of riparian zones for non-point source pollution control in Chongming Island: Status, challenges, and perspectives. J. Cleaner Prod 2020, 244, 118804.

[61] USDA. A Landowner’s Guide to the Wetlands Reserve Program (WRP); United States Department of Agriculture (USDA): Warwick, RI, USA, 2012.

[62] USDA. Conservation Reserve Program (CRP); United States Department of Agriculture (USDA): Warwick, RI, USA, 2019.

[63] USDA. Conservation Reserve Enhancement Program. Available online: https://www.fsa.usda.gov/programs-and-services/conservation-programs/conservation-reserve-enhancement/index (accessed on 7 May 2021).

